# The whole profiling and competing endogenous RNA network analyses of noncoding RNAs in adipose-derived stem cells from diabetic, old, and young patients

**DOI:** 10.1186/s13287-021-02388-5

**Published:** 2021-05-29

**Authors:** Sen Ren, Hewei Xiong, Jing Chen, Xiaofan Yang, Yutian Liu, Jiahe Guo, Tao Jiang, Zhao Xu, Meng Yuan, Yang Liu, Nan Zhou, Hongrui Chen, Wenqing Li, Hans-Günther Machens, Zhenbing Chen

**Affiliations:** 1 Department of Hand Surgery, Union Hospital, Tongji Medical College, Huazhong University of Science and Technology, No. 1277 Jiefang Avenue, Wuhan, 430022 China; 2 Department of Pathology, Union Hospital, Tongji Medical College, Huazhong University of Science and Technology, Wuhan, China; 3 Department of Hand and Foot Surgery, Huazhong University of Science and Technology Union Shenzhen Hospital, Shenzhen, Guangdong China; 4 Department of Plastic and Hand Surgery, Technical University of Munich, Munich, Germany

**Keywords:** Adipose-derived stem cell, Noncoding RNA, Competing endogenous RNA, Diabetes, Aging

## Abstract

**Background:**

Mesenchymal stem cells including adipose-derived stem cells (ASCs) have a considerable potential in the field of translational medicine. Unfortunately, multiple factors (e.g., older age, co-existing diabetes, and obesity) may impair cellular function, which hinders the overall effectiveness of autologous stem cell therapy. Noncoding RNAs—including microRNAs (miRNAs), long ncRNAs (lncRNAs), and circular RNAs (circRNAs)—have been shown to play important roles in stem cell biology. However, the overall diabetes-related and aging-related expression patterns and interactions of these RNAs in ASCs remain unknown.

**Method:**

The phenotypes and functions of ASCs isolated from diabetic (D-ASCs), old (O-ASCs), and young (Y-ASCs) donors were evaluated by in vitro assays. We conducted high-throughput RNA sequencing (RNA-seq) in these ASCs to identify the differentially expressed (DE) RNAs. Gene ontology (GO), Kyoto Encyclopedia of Genes and Genomes (KEGG) pathway, and protein-protein interaction (PPI) analyses were performed to investigate mRNAs with significant differences among groups. The lncRNA- or circRNA-associated competing endogenous RNA (ceRNA) networks were constructed based on bioinformatics analyses and real-time polymerase chain reaction (RT-PCR) results. The miR-145-5p mimics were transfected into O-ASCs and verified by PCR.

**Results:**

ASCs from diabetic and old donors showed inferior migration ability and increased cellular senescence. Furthermore, O-ASCs have decreased capacities for promoting endothelial cell angiogenesis and fibroblast migration, compared with Y-ASCs. The DE miRNAs, mRNAs, lncRNAs, and circRNAs were successfully identified by RNA-seq in O-ASCs vs. Y-ASCs and D-ASCs vs. O-ASCs. GO and KEGG analyses demonstrated that DE mRNAs were significantly enriched in aging and cell senescence terms separately. PPI networks revealed critical DE mRNAs in the above groups. RNAs with high fold changes and low *p* values were validated by PCR. ceRNA networks were constructed based on bioinformatics analyses and validated RNAs. Additionally, the lncRNA RAET1E-AS1–miR-145-5p–WNT11/BMPER axis was validated by PCR and correlation analyses. Finally, the overexpression of miR-145-5p was found to rejuvenate O-ASCs phenotype and augment the functionality of these cells.

**Conclusion:**

Our research may provide insights regarding the underlying mechanisms of ASC dysfunction; it may also offer novel targets for restoring therapeutic properties in ASCs.

**Supplementary Information:**

The online version contains supplementary material available at 10.1186/s13287-021-02388-5.

## Background

Stem cell-based therapies provide an alternative option for tissue regeneration and have been widely studied for the treatment of various human diseases in the 21st century. The most recent clinical successes involving limbal stem cells in corneal restoration and transgenic stem cells for epidermis regeneration have been compelling [1, 2], which presumably reflect the considerable potential of this field. However, most of these studies remain in preclinical or clinical trial stages. Although substantial investments have been made, clinical applications remain limited because of the poor efficacy and safety characteristics [3]. Notably, there are certain obstacles we must concern when applying stem cells to treat diseases. First, there is a risk of allosensitization when transplanting allogenic stem cells [4]. Second, intravascular stem cell therapy may trigger instant blood-mediated inflammatory reaction, as well as thrombosis and embolization [5]. Third, the hostile host microenvironment hinders stem cell vitality and functionality [6]. Finally, the therapeutic effect of stem cells can be easily impaired by physical conditions within donors (e.g., high age, diabetes, obesity, and chronic kidney disease) [7–9]. Given the high morbidity of diabetes and the rapidly aging global population, the donor condition merits extensive consideration.

Stem cells dysfunction causes localized failed tissue maintenance and regeneration, while limiting overall effectiveness in repairing distant tissues and organs [8, 9]. Adipose-derived stem cells (ASCs) have been recognized as a promising tool for the treatment of many disorders (e.g., nerve injury, skin wound healing, cardiac diseases, and autoimmune disorders) [10–12]. ASC functions are primarily mediated by their paracrine factors such as growth factors, cytokines, and extracellular vesicles [13, 14]. However, previous studies demonstrated that diabetic or aged ASCs had reduced capacities to promote neovascularization and skin wound healing [15, 16]. Other studies showed that diabetes and aging led to the upregulation of inflammatory markers and changes in ASC immunomodulatory properties [17, 18]. Furthermore, ASCs isolated from donors with diabetes showed impaired effects on the treatment of critical limb ischemia and diabetes-related complications [19, 20]. These cells showed inferior proliferative and proangiogenic capacities, as well as increased intracellular reactive oxygen species (ROS) accumulation; they also exhibited a pro-inflammatory secretome and senescence-associated phenotype, compared with ASCs from normal donors [21–23]. Thus, it is important to consider the negative influences of diabetes and aging on ASCs before implantation. Thus far, the precise molecular mechanisms of these alterations and impairments in the niche remain largely unknown. Identification of the underlying mechanisms may provide opportunities to reverse ASC malfunctions and achieve improved stem cell-based therapies.

Regulatory non-coding RNAs—including microRNAs (miRNAs), long non-coding RNAs (lncRNAs), and circular RNAs (circRNAs)—have been demonstrated to play essential roles in many cellular processes and influence various human diseases [24]. Recent studies have shown that these RNAs extensively participate in the proliferation, migration, differentiation, quiescence, and senescence of stem cells [25, 26]. miRNAs are small non-coding RNAs (22-26 nucleotides) that can repress gene expression post-transcriptionally [27]. Emerging evidences elucidated that diabetes and aging manipulated the expression levels of some miRNAs in stem cells, which might impair their functionalities [22, 28]. Moreover, modifying the levels of miR-122 [29], miR-5591-5p [30], and miR-34a-5p [31] could enhance the therapeutic efficacies of ASCs against liver fibrosis, diabetic wound healing, and ischemic myocardial infarction, respectively. lncRNAs are a cluster of transcripts with lengths greater than 200 nucleotides, which can function as signals, decoys, guides, and scaffolds [32]. Li et al. [33] found that the overexpression of lncRNA Bmncr could reverse aging-related damage in bone marrow mesenchymal stem cells (BMMSCs) and promote their ability to engage in bone formation. circRNAs are covalently closed, endogenous transcripts that function by regulating transcription and protein production, by interfering with splicing or by translating themselves [34]. Recent studies have demonstrated the important roles of circRNAs in aging, as well as various cancers and diabetes. Moreover, circRNAs and lncRNAs can function as competing endogenous RNAs (ceRNAs), which compete with miRNAs for binding to mRNAs, thus promoting the translation of target mRNAs [35]. ceRNA networks have been implicated in stem cell function [36]. Thus, the mechanism by which non-coding RNAs affect the therapeutic capacities of ASCs in relation to diabetes and aging warrants comprehensive investigation.

In this study, we conducted high-throughput RNA sequencing (RNA-seq) in ASCs isolated from type 2 diabetic, old and young patients to systematically explore dysregulated miRNAs, mRNAs, lncRNAs and circRNAs. To the best of our knowledge, this is the first establishment of lncRNA- and circRNA-associated ceRNA networks for simultaneous interpretation of poor ASC function in the context of diabetes and aging. Furthermore, we demonstrated that the lncRNA RAET1E-AS1–miR-145-5p–WNT11/BMPER axis may play a vital role in regulating ASC senescence and function. Therefore, we presume that this study will provide a theoretical basis and novel methods to restore the function of ASCs, thus promoting future ASC-based therapies.

## Methods and materials

### Cell culture

ASCs were isolated and incubated using our previously described protocols [11]. ASCs were cultured in Dulbecco’s modified Eagle’s medium (DMEM, Gibco, USA) supplemented with 10% fetal bovine serum (FBS, Serapro, USA) and 1% penicillin/streptomycin. ASCs were identified and characterized using a flow cytometric analysis and multi-lineage differentiation method, in accordance with previous published articles [11, 13, 37]. The patients under skin flap operations were selected from hand surgery department of Wuhan union hospital at the Tongji Medical College of Huazhong University of Science and Technology. These donors were consented at first, and then assigned into three groups: young group (Y-ASCs), old group (O-ASCs), and diabetic group (D-ASCs). The detailed information of these groups was described in Table 1. Human umbilical vein endothelial cells (HUVECs) (#GDC166, CCTCC) were obtained from the China Center for Type Culture Collection (CCTCC, Wuhan, China). Human foreskin fibroblasts were isolated and cultured following our previous protocols [11].

**Table 1:** Basic characteristics of different groups included in the study

Variables	Young donors	Old donors	Diabetic donors	*p* value (Y/O)	*p* value (O/D)
Number	14	15	8	NA	NA
Age Range	8.14 ± 2.93 [3, 12]	54.20 ± 3.95 [49, 61]	56.88 ± 8.01 [48, 71]	< 0.001*** NA	0.29 NA
BMI	19.13 ± 3.76	22.02 ± 2.72	23.71 ± 2.33	0.04*	0.21
SBP	115 ± 12.61	126.54 ± 8.64	130.42 ± 20.19	0.03*	0.55
DBP	75 ± 7.98	80.00 ± 6.40	82.71 ± 12.41	0.14	0.52
FBG	4.87 ± 0.37	5.00 ± 0.72	9.02 ± 2.52	0.60	< 0.001***
HbA1c	NA	NA	10.21 ± 2.75	NA	NA
TG	1.15 ± 0.52	1.53 ± 0.85	1.35 ± 0.66	0.29	0.59
TC	3.68 ± 0.65	4.44 ± 0.85	4.21 ± 1.31	0.20	0.71
HDL	1.23 ± 0.22	1.19 ± 0.33	0.89 ± 0.19	0.83	0.13
LDL	1.98 ± 0.67	2.65 ± 0.75	2.71 ± 1.33	0.01*	0.92
Cr	37.00 ± 5.42	51.08 ± 16.98	74.20 ± 11.46	0.02*	0.009**
BUN	3.89 ± 1.21	4.13 ± 0.91	4.84 ± 1.28	0.40	0.19

Diabetic donors: donors with type 2 diabetes; range: minimum age, maximum age; BMI (kg/m^2^): body mass index; SBP (mm Hg): systolic blood pressure; DBP (mm Hg): diastolic blood pressure; FBG (mmol/L): fasting blood glucose; HbA1c (%): glycated hemoglobin; TG (mmol/L): triglyceride; TC (mmol/L): total cholesterol; HDL-C (mmol/L): high-density lipoprotein cholesterol; LDL-C (mmol/L): low-density lipoprotein cholesterol; Cr (μmol/L): creatinine; BUN (mmol/L): blood urea nitrogen; NA: not available. Data are expressed as mean ± SD and were statistically analyzed by the un-paired Student’s *t* test. *p< 0.05, **p< 0.01, ***p< 0.001

### ASC proliferation and migration

ASCs at passage 4 from the above groups were grown in 96-well plates (5000 cells per well) for 24 h. After 2 h incubation with the EdU, the proliferation rates of ASCs from each group were evaluated with Cell-Light EdU Apollo In Vitro Kit (Ribobio, Guangzhou, China).

The 24-well Transwell Chamber (8.0 μm pore size, Corning, USA) was used for assessing the migration ability of ASCs from above groups. Briefly, ASCs (20,000 cells/well) suspended in DMEM without serum were added to the upper compartment, and then incubated in complete culture medium containing 10% FBS for 24 h. Then, ASCs migrated to the bottom surface were stained with crystal violet staining (Solarbio, Beijing, China) and counted under microscopy.

### Senescence-associated β-galactosidase staining

Senescence-associated β-galactosidase staining was performed in accordance with a previously published method [38]. ASCs (10,000 cells/well) at passage 4 from above groups were seeded in 48-well plates. After incubation for 3 days, the activity of senescence-associated β-Galactosidase was detected using Senescence β-gal Staining kit (#9860, CST) according to the manufacturer’s instruction.

### Wound scratch and tube formation

ASCs from each group were grown in six-well plates (2 × 10^5^ cells per well) for 2 days. The complete culture medium was then replaced with serum-free DMEM. After another day incubation, the medium was collected and considered as the conditioned medium.

Human fibroblasts (5 × 10^4^ cells per well) were seeded in 12-well plates and cultured until 100% confluence. Then, the monolayer was scored with a 200-μl sterile pipette tip. After that, the culture medium was replaced with conditioned media from above groups and maintained for 24 h. The cells were then stained with crystal violet and counted.

HUVECs (2 × 10^4^ cells per well) suspended in conditioned media were seeded in 96-well plates previously coated with 50 μl Matrigel Basement Membrane Matrix (BD Biosciences, USA). After 4 h incubation, tube formation was detected with the microscopy.

### RNA-seq

ASCs at passage 3 were used for RNA extraction and sequencing. Standard cDNA libraries were conducted and sequenced using the BGISEQ-500 platform and Illumina HiSeq 4000 platform (BGI-Shenzhen, China). The sequence data were filtered to get clean data, and then mapped to the reference genome with Bowtie2 [39] (version 2.2.5). Further, differential expression analysis was performed using the DEGseq [40]. The expected fragments per kilobase of transcript per million fragments sequenced (FPKM) [41] was used to determine the expression level of mRNAs, lncRNAs and circRNAs. The expression level of miRNAs was determined by the transcript per million (TPM) [41]. The significantly dysregulated RNAs must meet the following criteria: (1) fold changes ≥ 2 or ≤ − 2, (2) *p* and *q* value < 0.001, and (3) TPM value ≥ 10 for miRNA or FPKM value ≥ 0.1 for other RNAs. Detailed RNA-seq data have been deposited at Gene Expression Omnibus (http://www.ncbi.nlm.nih.gov/geo; GSE174502).

### RT-PCR

RT-PCR was performed in accordance with a previously published method [42]. Total RNA was isolated with miRNeasy Mini Kit (Qiagen, Germany) according to the manufacturer’s instructions. MiRNAs and other RNAs were reversely transcribed into cDNA using the Mir-X™ miRNA First-Strand Synthesis kit (#RR638315, TaKaRa) and Prime-Script® RT reagent Kit (#RR047A, TaKaRa) separately. Real-time PCR was performed on the StepOnePlus™ platform (Applied Biosystems, USA) using TB Green® Premix Ex Taq™ II kit (#RR820A, TaKaRa). The primer sequences were presented in Tables S2, S3, S4, S5. The relative expression levels of targeted genes were calculated using the 2^−ΔΔCt^ method and normalized to β-actin, gapdh, or u6.

### Western blotting

Western blotting was performed in accordance with a previously published method [43]. Total proteins were extracted by ripa lysis buffer with proteinase inhibitor (Roche, Switzerland). In total, 30 μg aliquots of protein were separated in 10% SDS-PAGE gels, then transferred onto PVDF membranes (Millipore, USA). After 1.5 h of blocking with 5% w/v nonfat dry milk buffer, the membrane was incubated overnight with primary antibodies for fibronectin (1:1000, #A12932, ABclonal, China), cyclin D1 (1:1000, #A11022, ABclonal, China), cyclin A1 (1:1000, #ab206746, Abcam), NANOG (1:1000, #14295-1-AP, Proteintech, China), OCT4 (1:1000, #11263-1-AP, Proteintech, China), p21 (1:1000, #10355-1-AP, Proteintech, China), and GAPDH (1:1000, #10494-1-AP, Proteintech, China). Then, the membrane was incubated with secondary antibodies (1:5000, Aspen, China) and exposed to X-ray film (UVP, USA).

### ceRNA and PPI network analysis

The significant differentially expressed mRNAs and ncRNAs between each group were used for ceRNA network constructions. The RNAs which could be predicted by at least two of these databases (RNAhybrid [https://bibiserv.cebitec.uni-bielefeld.de/rnahybrid], miRanda [http://www.microrna.org/microrna/home.do] and TargetScan [http://www.targetscan.org]) were considered as the miRNA targets. The sequences of mRNAs, lncRNAs, and circRNAs were screened to get the potential MREs. Protein-protein interaction (PPI) analysis of differentially expressed mRNAs was based on the STRING database (https://string-db.org). These networks were illustrated using Cytoscape 3.7.1. The degree centrality of involved genes was calculated by Cytoscan.

### GO and KEGG enrichment analyses

The differentially expressed mRNAs were analyzed by Gene Ontology (GO) database and Kyoto Encyclopedia of Genes and Genomes (KEGG) pathway database. The GO categories (http://geneontology.org) were used for defining the molecular function and biological process involved of candidate genes. The biological function of these genes was further annotated by KEGG database (http://www.genome.jp/kegg). We used the hypergeometric distribution test to find out significantly enriched gene sets. A *p* value < 0.05 was considered statistically significant.

### Cell transfection

The mimic and inhibitor of hsa-miR-145-5p were designed and synthesized by Ribobio (Guangzhou, China). ASCs were transfected using riboFECT™CP Reagent according to the manufacturer’s instruction. After 48 h of incubation, ASCs were harvested for subsequent experiments. The sequences of these oligonucleotides are described in Table S5.

### Statistics

All statistical analysis was conducted using Graph Pad prism v 7.0 software. All data were expressed as the mean ± SEM. Unpaired Student’s *t* test was used for evaluating the statistical discrepancy between two groups. For group ≥ 3, one-way ANOVA with Bonferroni post hoc test was employed. The Pearson correlation coefficient was used to calculate the expressional correlation of two RNAs. Statistical significance was set at *p* or *q* < 0.05.

## Results

### ASCs from diabetic or old donors showed inferior proliferation and migration ability, exhibited a senescent phenotype

**Fig. 1:**
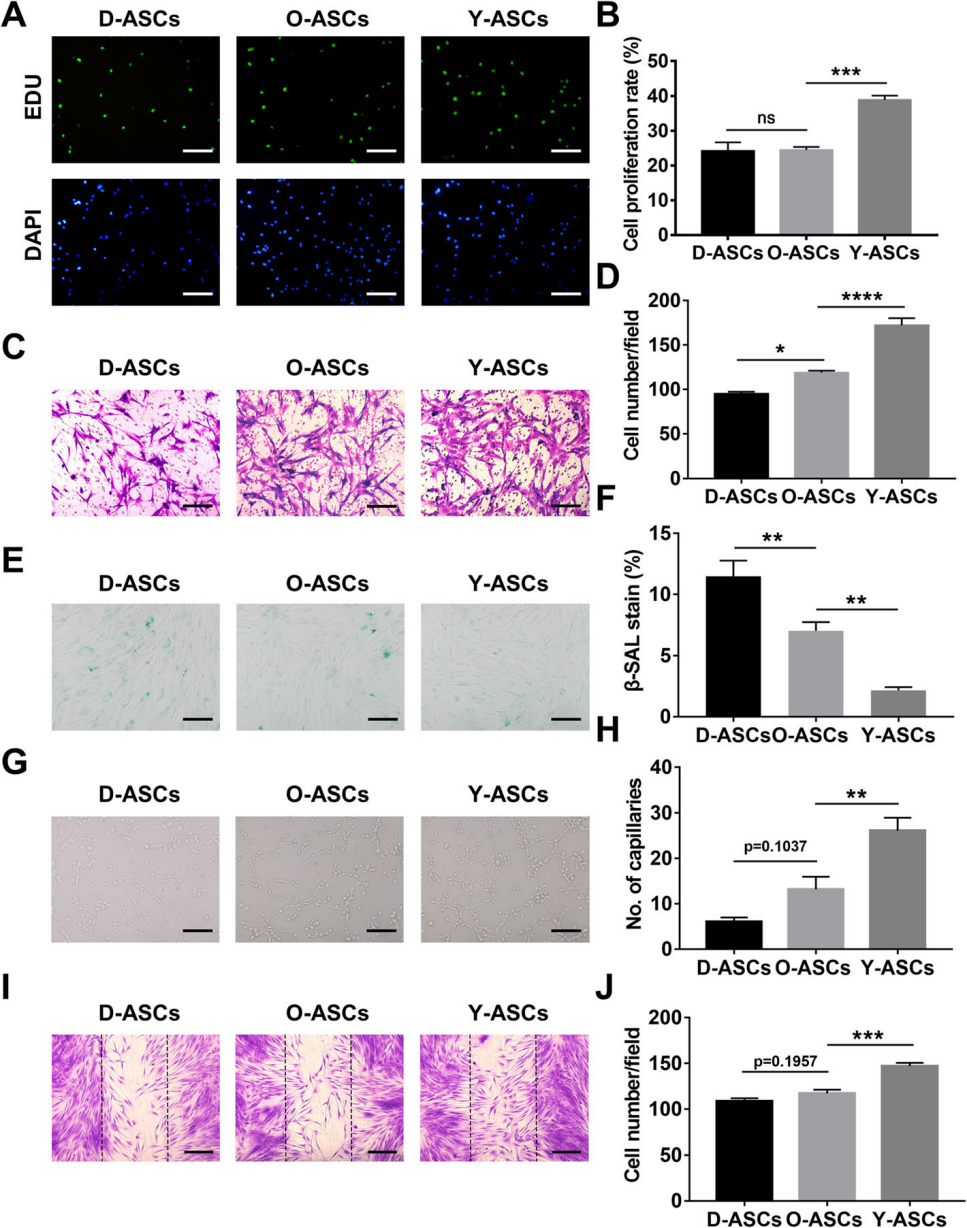
Evaluation of the phenotype and function of ASCs isolated from diabetic, old, and young patients. **a** EdU assay analysis of the proliferation rate of ASCs from those three groups. The proliferative cells and cellular nuclei were stained with green and blue colors. Scale bar 50 μm. **c** Images of migratory ASCs from those three groups. Scale bar 50 μm. **e** β-gal Staining assay analysis of the senescent rate of ASCs from those three groups. The senescent cells were stained with the blue color. Scale bar 50 μm. **g** Images of tube formations in HUVECs treated with conditioned media of ASCs from those three groups. Scale bar 50 μm. **i** Images of migratory fibroblasts given above treatments. Scale bar 100 μm. **b**, **d**, **f**, **h**, and **j** Qualified data shown in **a**, **c**, **e**, **g**, and **i** separately. *N* = 5. *p< 0.05, **p< 0.01, ***p< 0.001, ****p< 0.0001

To identify whether diabetes and aging affected ASC phenotype, a series of fundamental experiments was conducted. First, the EdU assay showed that the proliferation rate of ASCs from old donors was 24.48%, which was significantly lower than the proliferation rate of ASCs from young donors (38.88%) (Fig. 1a, b). Notably, diabetic conditions did not reduce the proliferation rate of ASCs (Fig. 1a, b). Next, Transwell assays revealed that the migration ability of ASCs was best in the young group, followed by the old group; the worst ability was observed in the diabetic group (Fig. 1c, d). Last, β-galactosidase staining showed that the rate of senescent stem cells in diabetic group was significantly higher in the diabetic group than in the old group, while ASCs in the young group exhibited the lowest number of senescent cells (Fig. 1e, f). Overall, diabetes and aging both have detrimental effects on ASC phenotype.

### Diabetic conditions and aging impaired the capacities of ASCs for modulating fibroblast and HUVEC functions

Given the above results, we investigated whether diabetic conditions and aging impaired the therapeutic abilities of ASCs. Thus, we evaluated the differences in ASCs from these three groups in terms of their abilities to optimize HUVEC and fibroblast functions. In vitro tube formation assays showed that HUVECs treated with conditioned media from Y-ASCs exhibited twofold increases in the number of closed tubular structures, compared with HUVECs treated with conditioned media from O-ASCs (Fig. 1g, h). Moreover, the number of tubular structures was twofold greater in the O-ASC group than in the D-ASC group, but this difference was not statistically significant (Fig. 1g, h). Notably, wound scratch assays revealed that the migration of fibroblasts treated with conditioned media from Y-ASCs was significantly greater than the migration of fibroblasts treated with conditioned media from O-ASCs (Fig. 1i, j). Additionally, the number of migratory fibroblasts was slightly lower in the D-ASC group than in the O-ASC group (Fig. 1i, j). Therefore, old donor age has substantially deleterious effects on the abilities of ASCs to modulate cellular functions, while the presence of diabetes in donors may have a slight harmful effect on ASC function.

### Differential expression analyses: Y-ASCs vs. O-ASCs and D-ASCs vs. O-ASCs

**Fig. 2:**
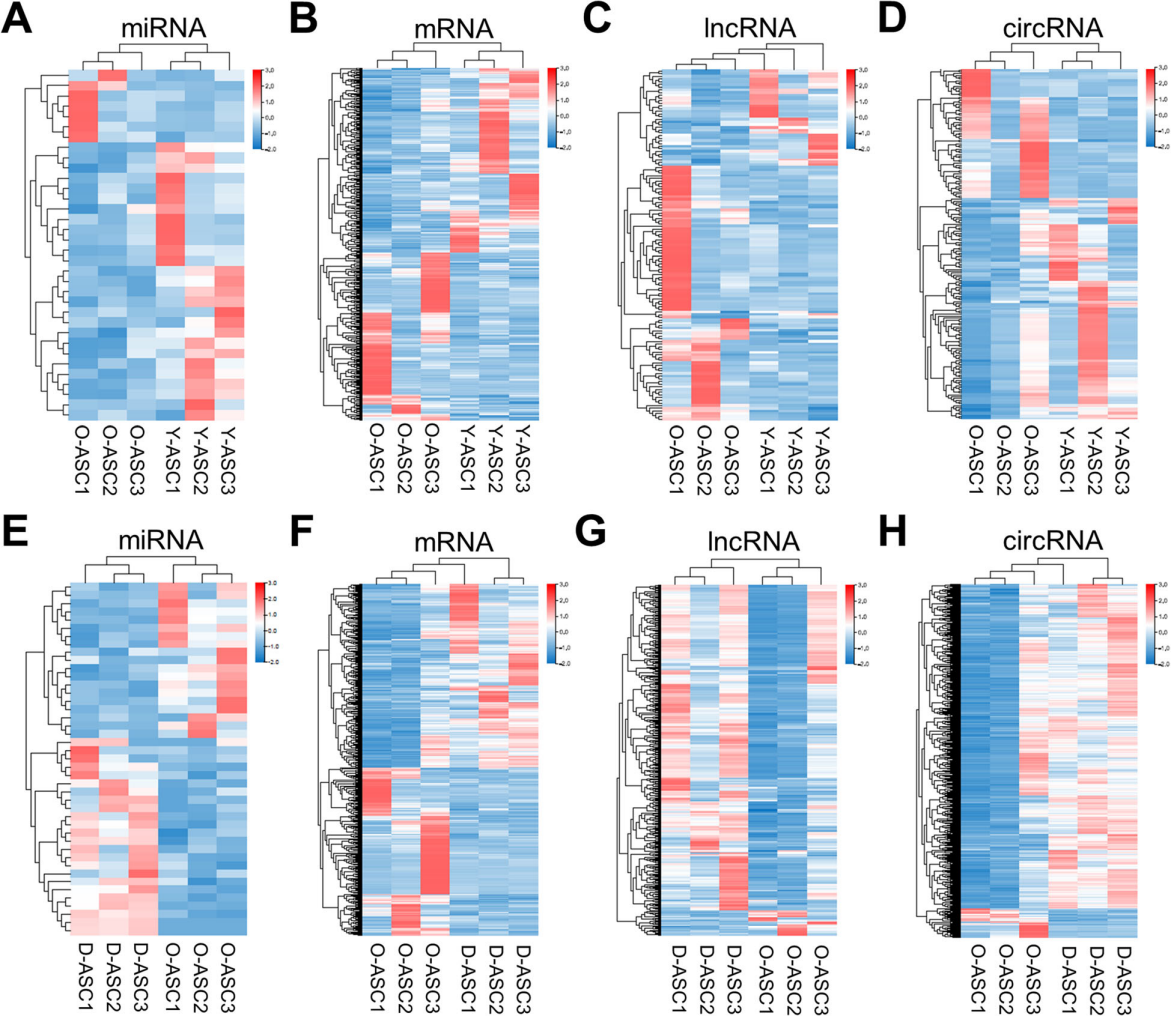
Expression profiles of RNAs. **a**–**d** The miRNAs, mRNAs, lncRNAs, and circRNAs profiles were shown in heatmap in O-ASCs vs. Y-ASCs. **e**–**h** The miRNAs, mRNAs, lncRNAs, and circRNAs profiles were shown in heatmap in O-ASCs vs. D-ASCs. *N* = 3

Following identification of the negative influences of diabetes and aging on ASC phenotype and function, we performed RNA-seq analysis on ASCs from these three groups to uncover the underlying mechanisms. The differentially expressed miRNAs, mRNAs, lncRNAs, and circRNAs from all three groups (*n* = 3) were illustrated using hierarchical clustering heat maps (Fig. 2). In total, 34 (27 up and seven down) differentially expressed miRNAs, 553 (291 up and 262 down) differentially expressed mRNAs, 132 (39 up and 92 down) differentially expressed lncRNAs, and 196 (123 up and 73 down) differentially expressed circRNAs were detected between Y-ASCs and O-ASCs (Fig. 2a–d, Data S1, S2, S3, S4). In this context, up indicates RNAs upregulated in Y-ASCs, compared with O-ASCs. Comparing D-ASCs with O-ASCs, we found 43 (24 up and 19 down) differentially expressed miRNAs, 926 (483 up and 443 down) differentially expressed mRNAs, 535 (496 up and 39 down) differentially expressed lncRNAs, and 1966 (1806 up and 160 down) differentially expressed circRNAs (Fig. 2e–h, Data S5, S6, S7, S8). In this context, up indicates RNAs upregulated in D-ASCs, compared with O-ASCs. Overall, we found that four miRNAs, 22 mRNAs, eight circRNAs were upregulated in O-ASCs, compared with D-ASCs; these RNAs were all upregulated in Y-ASCs, compared with O-ASCs (Data S9, S10, S11). Moreover, 15 mRNAs, 14 lncRNAs, and 17 circRNAs were downregulated in O-ASCs, compared with D-ASCs; these RNAs were all downregulated in Y-ASCs, compared with O-ASCs (Data S10, S11, S12).

### Functional enrichment analysis of differentially expressed mRNAs

**Fig. 3:**
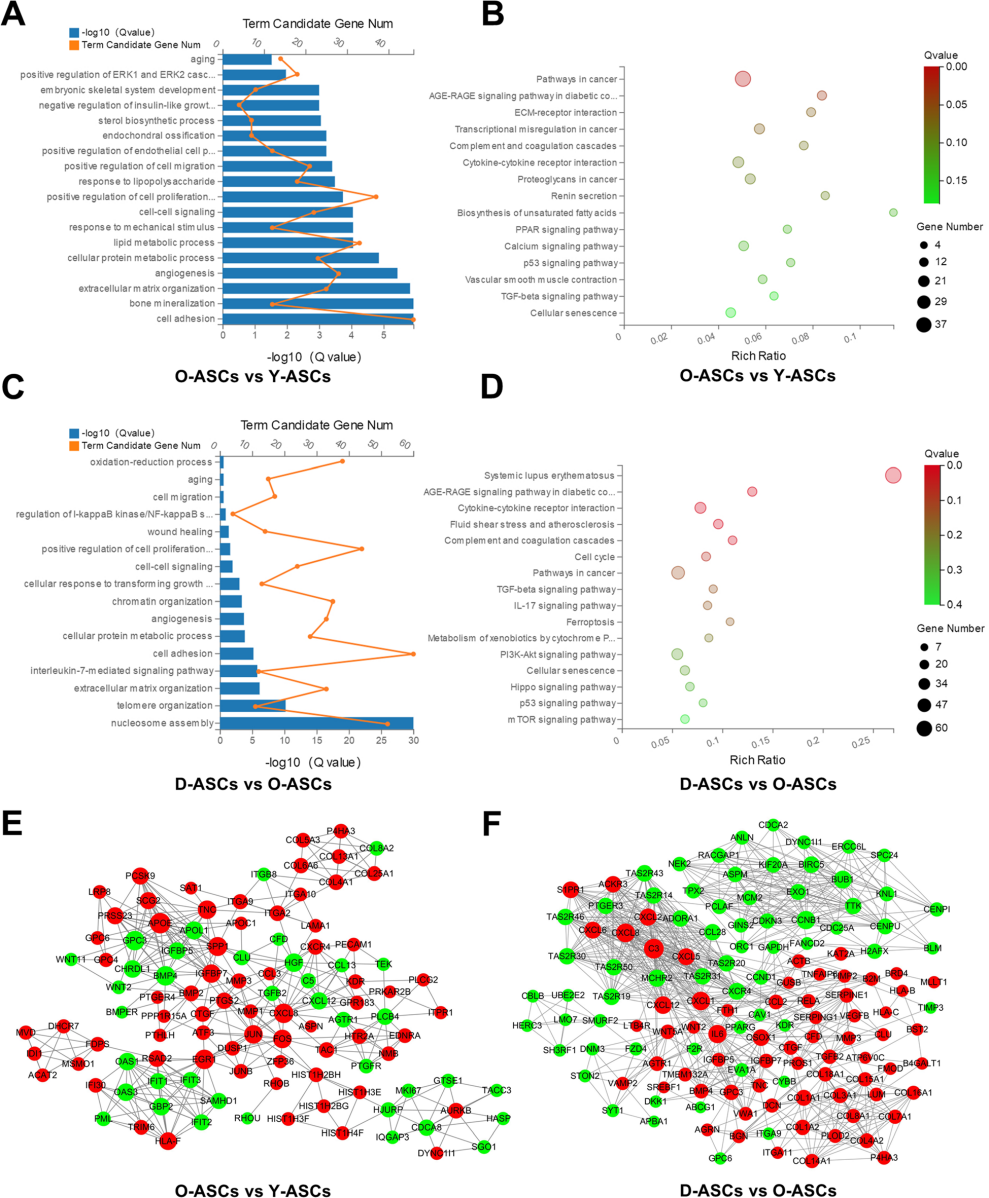
Bioinformatics analyses of the differentially expressed mRNAs. **a**, **b**, **e** GO, KEGG pathway, and PPI network analyses of the differentially expressed mRNAs in O-ASCs vs. Y-ASCs. **c**, **d**, **f** GO, KEGG pathway, and PPI network analyses of the differentially expressed mRNAs in O-ASCs vs. D-ASCs.

GO and KEGG analyses were conducted to investigate the biological functions of dysregulated mRNAs. Comparing Y-ASCs with O-ASCs, GO biological process analysis showed that 750 GO terms were significantly enriched (Data S13). Terms such as aging, positive regulation of cell proliferation, positive regulation of cell migration and angiogenesis were potentially associated with donor age-related ASC dysfunction (Fig. 3a). KEGG pathway analysis showed that 47 pathways were statistically enriched (Data S14). Among them, the cellular senescence, TGF-beta signaling, p53 signaling, and PPAR signaling pathways may be involved in impaired ASC functions (Fig. 3b). Comparing O-ASCs with D-ASCs, 553 GO terms were significantly enriched in GO biological process analysis (Data S15). Terms such as oxidation-reduction process, aging, cell migration, wound healing and angiogenesis may be associated with decreased efficacy in ASCs derived from patients with diabetes (Fig. 3c). KEGG pathway analysis revealed that 37 pathways were statistically enriched (Data S16). Among them, the AGE-RAGE signaling, TGF-beta signaling, p53 signaling, mTOR signaling, and cellular senescence pathways may be involved in the impaired functions of ASCs isolated from diabetic donors (Fig. 3d).

### PPI network construction

PPI networks were constructed to identify critical genes among the differentially expressed mRNAs. Comparing Y-ASCs with O-ASCs, the established network comprised 107 nodes and 373 edges (Fig. 3e). In this network, the top 15 genes with the highest core degree were APOE, BMP4, EGR1, GPC3, CXCL8, SPP1, FOS, HGF, TNC, IGFBP7, JUN, APOL1, GBP2, HLA-F, and OAS1 (Fig. 3e). Comparing O-ASCs with D-ASCs, the established network comprised 131 nodes and 783 edges (Fig. 3f). In this network, the top 15 genes with the highest core degree were C3, CXCL8, IL6, CXCL1, CCNB1, BUB1, CXCL12, MCHR2, CXCL2, CXCL6, CXCR4, CXCL5, QSOX1, S1PR1, and TAS2R31 (Fig. 3f).

### RT-PCR confirmation of differentially expressed miRNAs

**Fig. 4:**
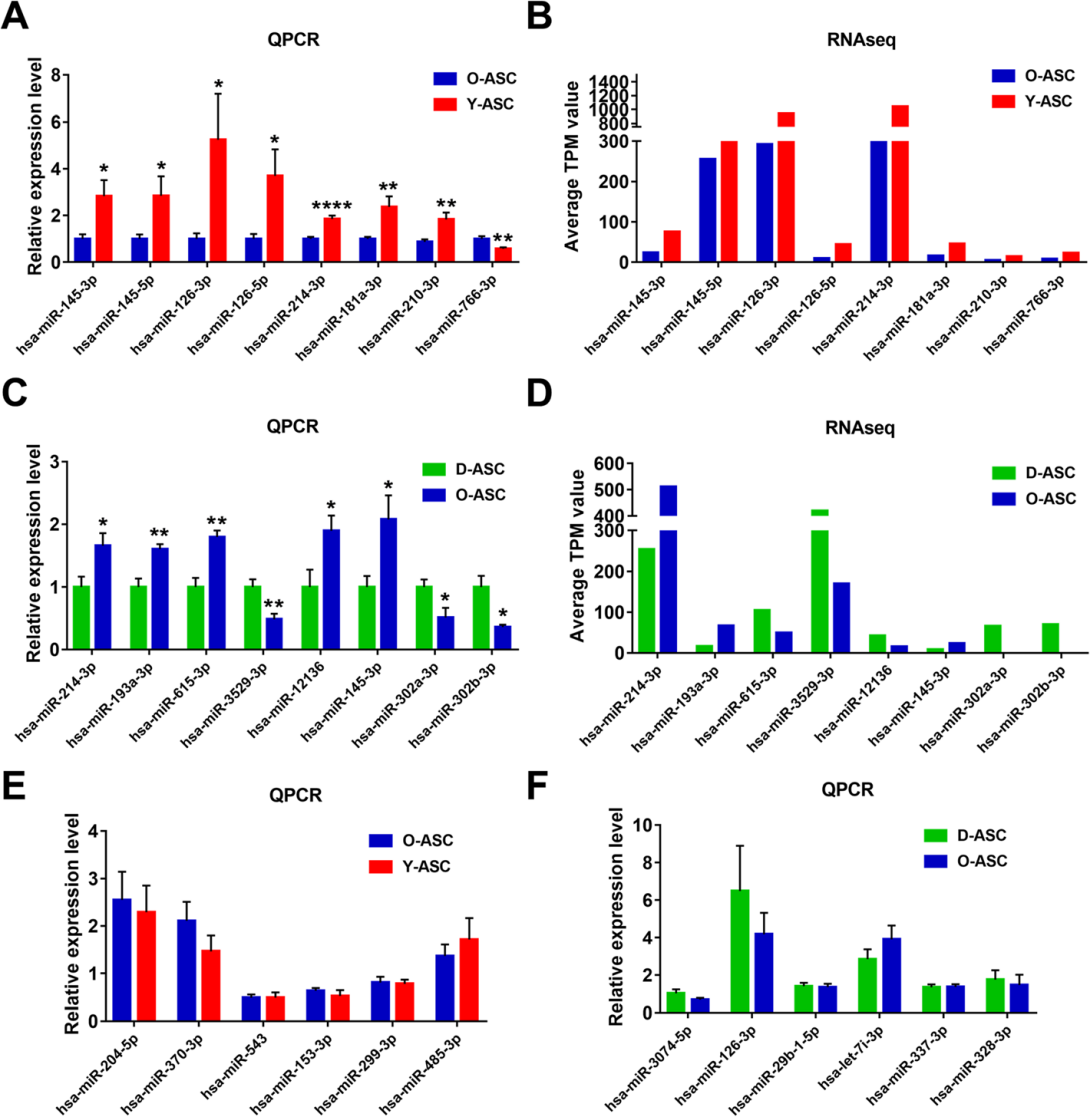
PCR analysis of the differentially expressed miRNAs. **a** PCR validation of the differentially expressed miRNAs in O-ASCs vs. Y-ASCs. *N* = 9. **b** RNA-seq profiles of the selected miRNAs in **a**. **c** PCR validation of the differentially expressed miRNAs in O-ASCs vs. D-ASCs. *N* = 5. **d** RNA-seq profiles of the selected miRNAs in **c**. miRNAs with no significant difference by PCR analysis were shown in **e**, **f**. *N* = 5. *p< 0.05, **p< 0.01, ***p< 0.001, ****p< 0.0001

We randomly selected 14 miRNAs to validate the reliability of RNA-seq data using RT-PCR. Comparing Y-ASCs with O-ASCs, PCR analysis showed that miR-145-3P, miR-145-5p, miR-126-3p, miR-126-5p, miR-214-3p, miR-181a-3p, and miR-210-3p were upregulated in Y-ASCs; these findings were consistent with the RNA-seq results (Fig. 4a, b). Furthermore, PCR analysis showed miR-766-3p was downregulated in Y-ASCs, which conflicted with the RNA-seq findings (Fig. 4a, b). Comparing D-ASCs with O-ASCs, PCR analysis showed that miR-214-3p, miR-193a-3p, and miR-145-3p were downregulated in D-ASCs, miR-3529-3p, miR-302a-3p, and miR-302b-3p were upregulated in D-ASCs; these findings were consistent with the RNA-seq results (Fig. 4c, d). However, PCR analysis of two miRNAs (miR-615-3p and miR-12136) showed findings that contrasted with the RNA-seq results (Fig. 4c, d). The remaining six miRNAs showed no significant difference between groups according to PCR analysis (Fig. 4e, f).

### Construction of circRNA– or lncRNA–miRNA–mRNA network

**Fig. 5:**
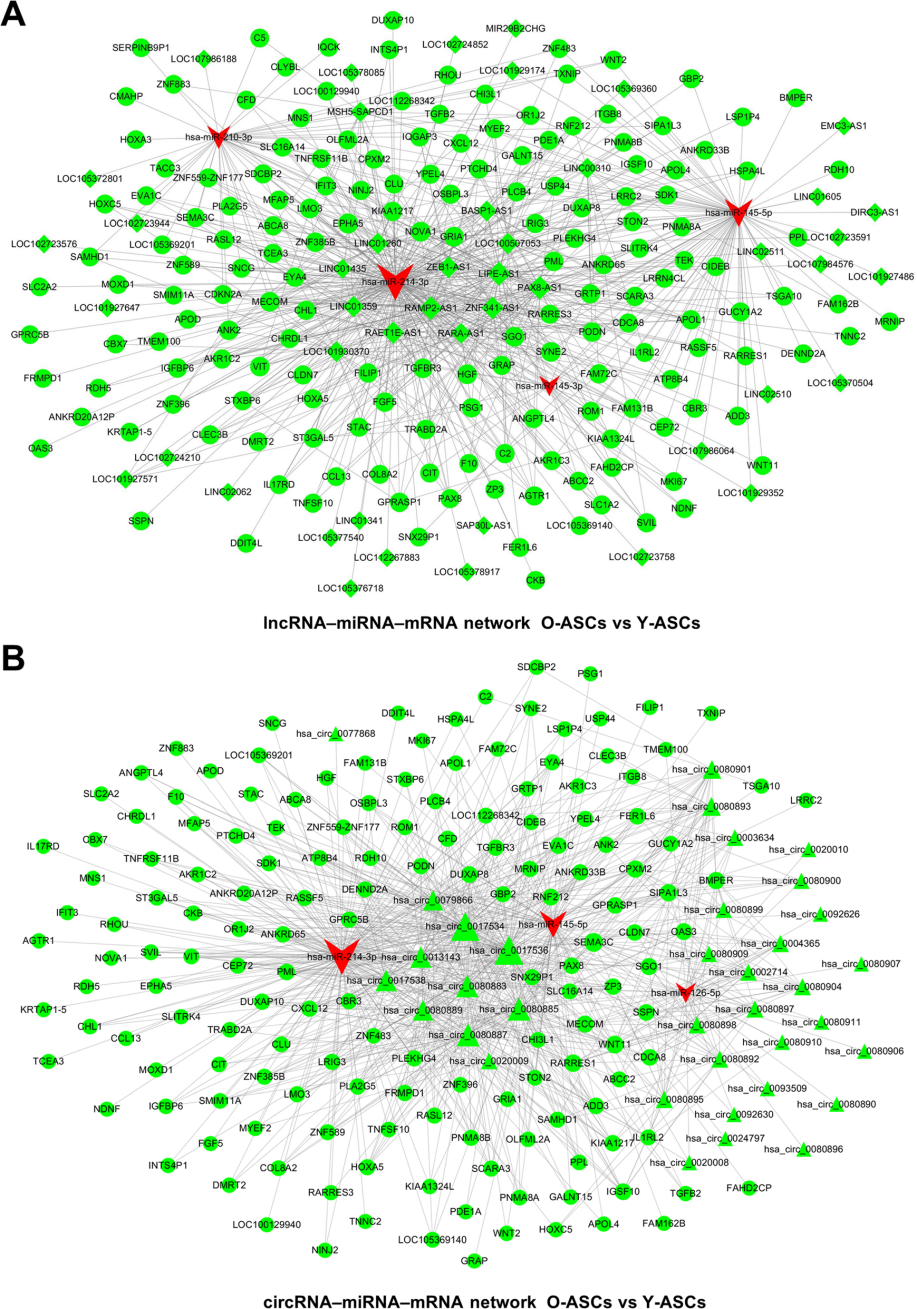
ceRNA network construction in O-ASCs vs. Y-ASCs. lncRNA (**a**)– and cirRNA (**b**)–miRNA–mRNA networks were constructed based on the differentially expressed lncRNAs, circRNAs, mRNAs, and validated miRNAs

**Fig. 6:**
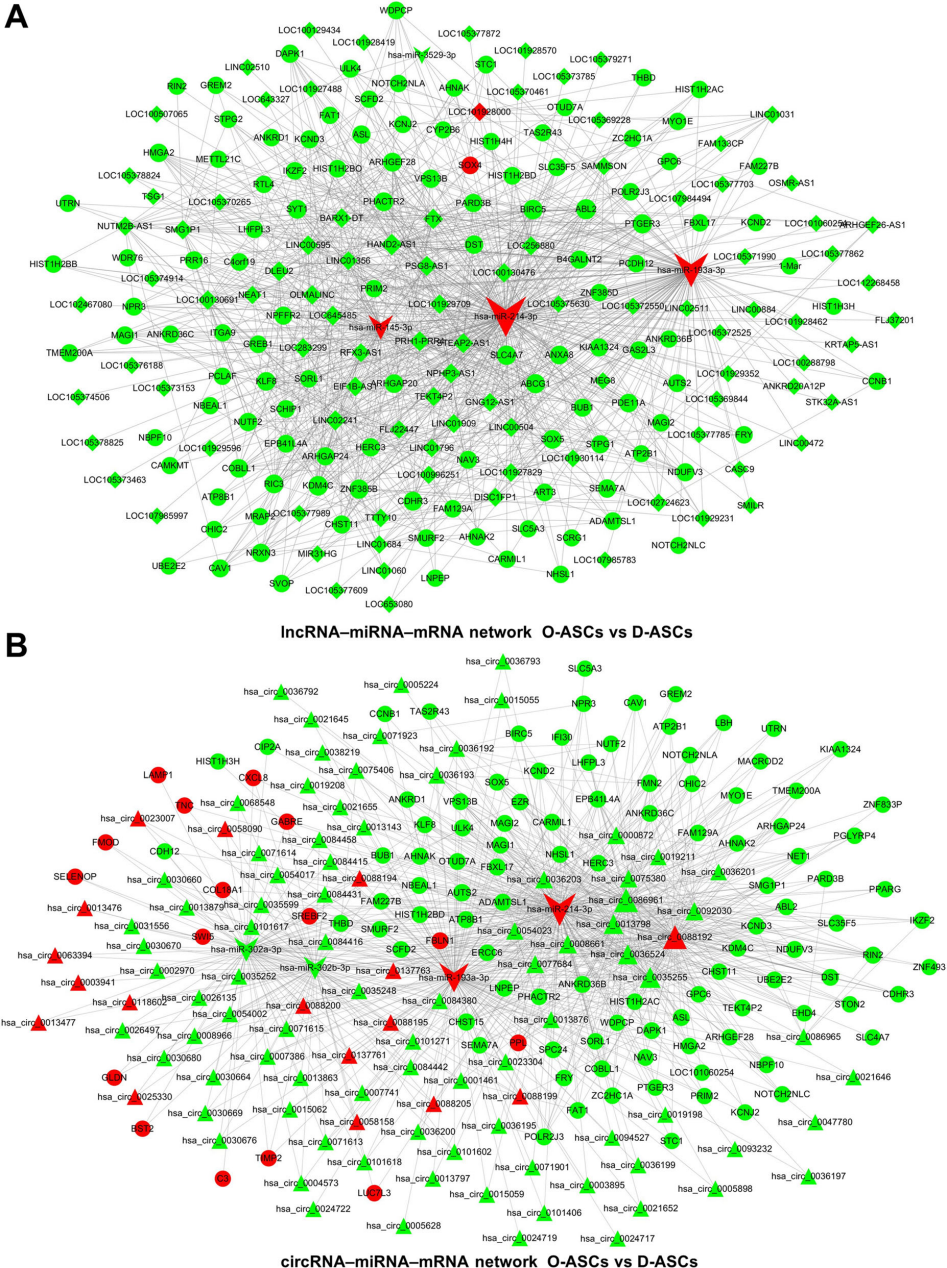
ceRNA network construction in D-ASCs vs. O-ASCs. lncRNA (**a**)– and cirRNA (**b**)–miRNA–mRNA networks were constructed based on the differentially expressed lncRNAs, circRNAs, mRNAs, and validated miRNAs

ceRNA networks were constructed based on the differentially expressed RNAs. miRNAs verified by PCR analysis, which exhibited expression patterns consistent with RNA-seq results, were selected as the cores of ceRNA networks. Comparing Y-ASCs with O-ASCs, the lncRNA-associated ceRNA network comprised four miRNAs, 171 mRNAs, and 46 lncRNAs (Fig. 5a); the circRNA-associated ceRNA network comprised three miRNAs, 160 mRNAs, and 36 circRNAs (Fig. 5b). Comparing D-ASCs with O-ASCs, the lncRNA-associated ceRNA network comprised four miRNAs, 118 mRNAs, and 97 lncRNAs (Fig. 6a); the circRNA-associated ceRNA network comprised four miRNAs, 119 mRNAs, and 103 circRNAs (Fig. 6b).

### RT-PCR confirmation of differentially expressed mRNAs, lncRNAs, and circRNAs

**Fig. 7:**
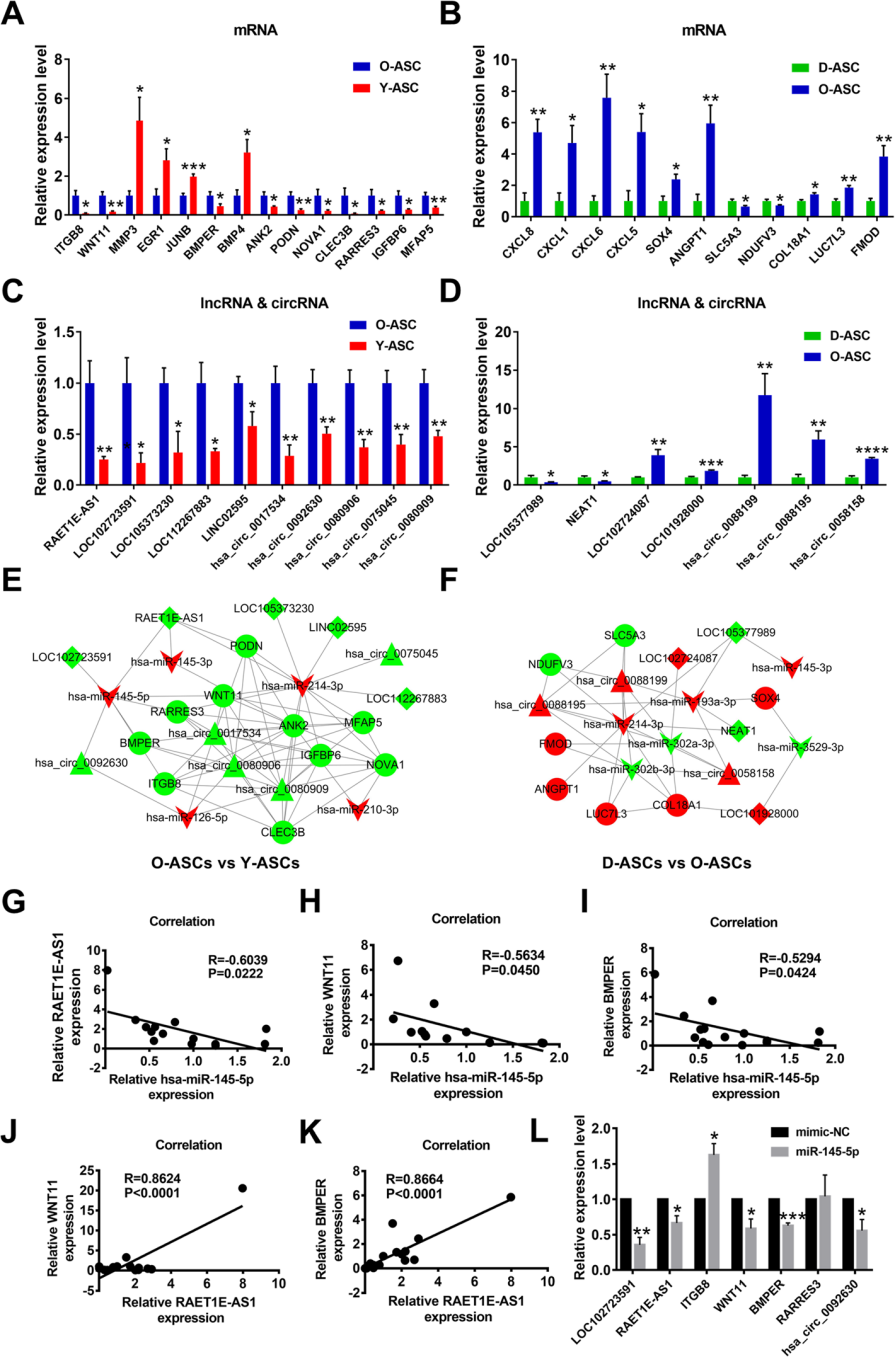
PCR analysis of RNAs in the above PPI and ceRNA networks, and construction of ceRNA sub-networks. **a**, **b** PCR validation of the selected mRNAs in O-ASCs vs. Y-ASCs and D-ASCs vs. O-ASCs. *N* = 5. **c**, **d** PCR validation of the selected lncRNAs and circRNAs in O-ASCs vs. Y-ASCs and D-ASCs vs. O-ASCs. *N* = 5. **e**, **f** Construction of ceRNA sub-networks based on the above PCR results. **g**–**i** Correlation analysis of the expression level of miR-145-5p with the expression levels of RAET1E-AS1, WNT11, and BMPER. *N* = 13-15. **j**, **k** Correlation analysis of the expression level of RAET1E-AS1 with the expression levels of WNT11 and BMPER. *N* = 15, 17. **l** PCR analysis of the expressional changes of RNAs in the ceRNA sub-network after miR-145-5p overexpression in ASCs. *p< 0.05, **p< 0.01, ***p< 0.001, ****p< 0.0001

mRNAs, lncRNAs, and circRNAs contained in the above PPI and ceRNA networks were selected for further RT-PCR analyses. Comparing Y-ASCs with O-ASCs, RT-PCR results showed that 14 mRNAs were significantly dysregulated (Fig. 7a). Among these dysregulated mRNAs, MMP3, EGR1, JUNB, and BMP4 were involved in the PPI network (Figs. 3e and 7a); ANK2, PODN, NOVA1, CLEC3B, RARRES3, IGFBP6, and MFAP5 were involved in the ceRNA network (Figs. 5a, b and 7a); and ITGB8, WNT11, and BMPER were involved in both PPI and ceRNA networks (Figs. 3e; 5a, b; and 7a). Comparing D-ASCs with O-ASCs, 11 mRNAs were confirmed to be differentially expressed by PCR analyses (Fig. 7b); of these, CXCL8, CXCL1, CXCL6, and CXCL5 were all upregulated in O-ASCs and involved in the PPI network (Figs. 3f and 7b). Furthermore, SOX4, ANGPT1, SLC5A3, NDUFV3, and LUC7L3 were involved in the ceRNA network (Figs. 6a, b and 7b); COL18A1 and FMOD were involved in both PPI and ceRNA networks (Figs. 3f; 6a, b; and 7b). Moreover, we found that five lncRNAs (RAET1E-AS1, LOC102723591, LOC105373230, LOC112267883, and LINC02595) and five circRNAs (hsa_circ_0017534, hsa_circ_0092630, hsa_circ_0080906, hsa_circ_0075045, and hsa_circ_0080909) were significantly upregulated in O-ASCs, compared with Y-ASCs (Fig. 7c). Additionally, PCR analysis revealed that two lncRNAs (LOC102724087 and LOC101928000) and three circRNAs (hsa_circ_0088199, hsa_circ_0088195, and hsa_circ_0058158) were significantly upregulated in O-ASCs, compared with D-ASCs (Fig. 7d). Conversely, two lncRNAs (LOC105377989 and NEAT1) were significantly downregulated in O-ASCs, compared with D-ASCs (Fig. 7d). Other RNAs without statistically significant differences between these groups are displayed in Figure S1A and B.

### Establishment of ceRNA sub-networks based on RNAs verified by PCR analyses

Based on the above PCR results, we constructed circRNA– and lncRNA–miRNA–mRNA sub-networks for further investigation. Comparing Y-ASCs with O-ASCs, the network included five miRNAs, 10 mRNAs, five lncRNAs, and five circRNAs (Fig. 7e). Comparing D-ASCs with O-ASCs, the network included six miRNAs, seven mRNAs, four lncRNAs, and three circRNAs (Fig. 7f). We randomly selected hsa-miR-145-5p for further analysis; it had ceRNA relationships with four mRNAs (ITGB8, WNT11, BMPER, and RARRES3), two lncRNAs (RAET1E-AS1 and LOC102723591), and one circRNA (hsa_circ_0092630) (Fig. 7e). Correlation analyses revealed that the expression levels of RAET1E-AS1, WNT11, and BMPER had significantly negative correlations with the expression level of hsa-miR-145-5p (Fig. 7g–i), while the other RNAs did not exhibit such relationships (Figure S1C, D). Furthermore, the expression level of RAET1E-AS1 had a significantly positive correlation with the expression levels of WNT11 and BMPER (Fig. 7j, k). Finally, we found that overexpression of has-miR-145-5p in ASCs could significantly downregulated the expression levels of WNT11, BMPER, RAET1E-AS1, LOC102723591, and hsa_circ_0092630 (Fig. 7l). Therefore, we speculated that the RAET1E-AS1–hsa-miR-145-5p–WNT11/BMPER network might play a vital role in the phenotype and function of ASCs.

### Overexpression of hsa-miR-145-5p improved the phenotype and function of ASCs, while its inhibition adversely affected the cells.

**Fig. 8:**
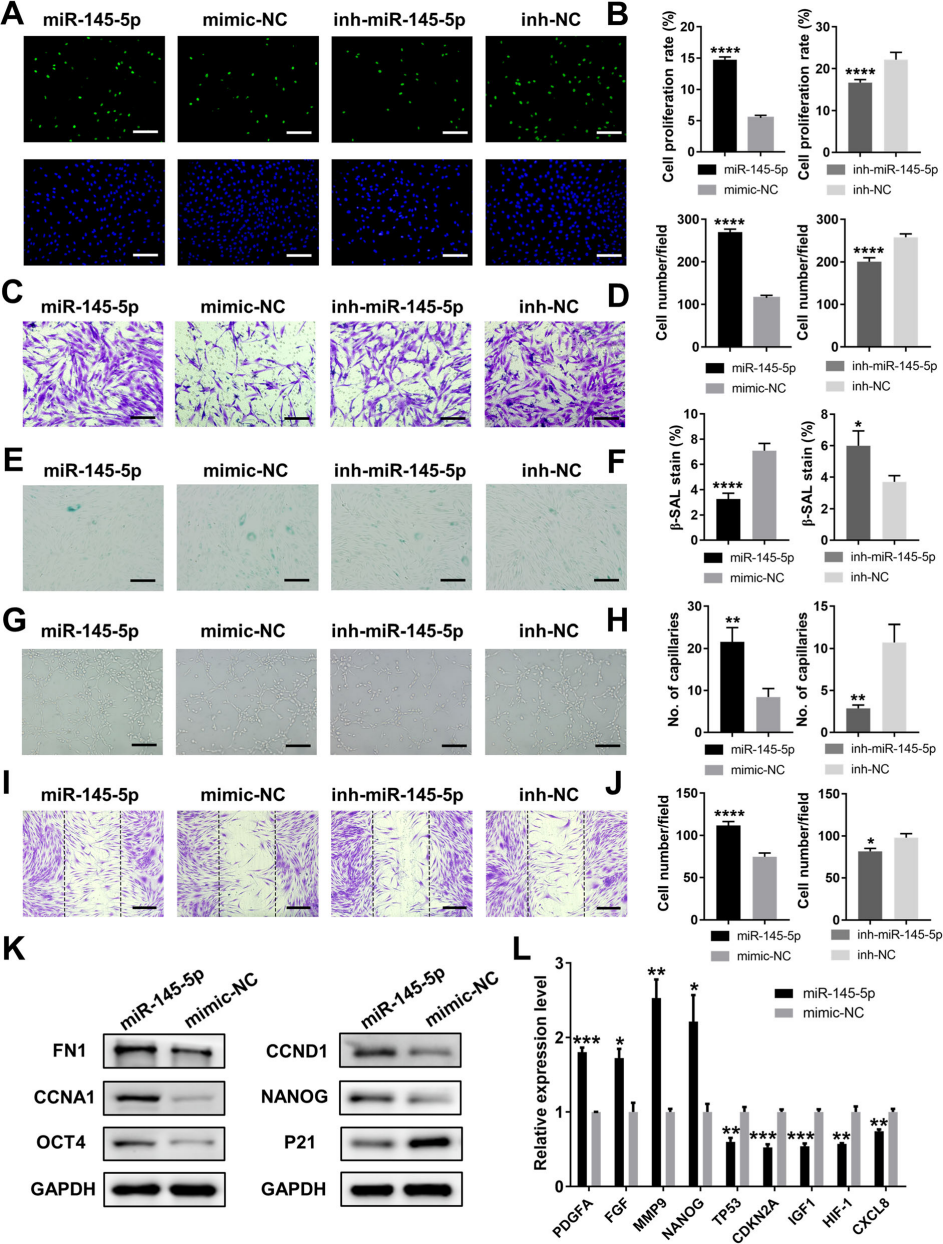
Overexpression of miR-145-5p rejuvenated O-ASCs and enhanced their functions. **a** The proliferation rate of ASCs treated with miR-145-5p, mimic-NC, inh- miR-145-5p, or inh-NC were measured by EdU assay. Scale bar 50 μm. **c** Images of migratory ASCs given above treatments. Scale bar 50 μm. **e** β-gal staining assay analysis of the senescent rate of ASCs given above treatments. Scale bar 50 μm. **g** Images of tube formations in HUVECs treated with conditioned media of ASCs given above treatments. Scale bar 50 μm. **i** Images of migratory fibroblasts given above treatments. Scale bar 100 μm. **b**, **d**, **f**, **h**, and **j** Qualified data shown in **a**, **c**, **e**, **g**, and **i** separately. **k** Western blot analysis of the proteins levels in ASCs after miR-145-5p overexpression. **l** PCR analysis of the genes expressions in ASCs after miR-145-5p overexpression. *p< 0.05, **p< 0.01, ***p< 0.001, ****p< 0.0001

To evaluate the characteristics of the RAET1E-AS1–hsa-miR-145-5–WNT11/BMPER axis, we modulated the expression level of hsa-miR-145-5p in ASCs from old donors to investigate changes in their phenotypes and functions. miR-145-5p mimic, mimic-NC, miR145-5p inhibitor, or inhibitor NC was transfected into ASCs separately, and transfection efficiency was evaluated by PCR analysis (Figure S2). EdU and Transwell assays showed that the proliferation and migration abilities of ASCs were promoted by more than twofold after hsa-miR-145-5p overexpression, while its inhibition significantly reduced these abilities (Fig. 8a–d). Furthermore, the number of β-galactosidase-stained cells was twofold less in the miR-145-5p group than in the mimic-NC group, while inhibition of miR-145-5p significantly increased the number of stained cells (Fig. 8e, f). Additionally, in vitro tube formation and wound scratch assays revealed that cellular supernatants of cultured ASCs with miR-145-5p overexpression could remarkably enhance the functions of HUVECs and fibroblasts, as shown by increases in closed tubular structures and migratory cells; the inhibition of miR-145-5p led to opposite results (Fig. 8g–j). Additionally, western blot analysis showed that overexpression of miR-145-5p in ASCs could significantly improve the expression of migration–associated protein FN1, proliferation–associated proteins CCNA1 and CCND1, and pluripotent markers NANOG and OCT4; it could decrease the expression of senescence–associated protein p21 (Fig. 8k). PCR analysis showed that overexpression of miR-145-5p in ASCs could significantly improve the expression of MMP9, NANOG, and growth factors PDGFA and FGF; it could decrease the expression of IGF1, HIF-1, CXCL8, and senescent markers TP53 and CDKN2A (Fig. 8l). Overall, the overexpression of miR-145-5p in ASCs could ameliorate the unsatisfactory phenotype and function of ASCs isolated from old donors.

## Discussion

Stem cells have an important role in the field of translational medicine, largely through the secretion of trophic factors rather than direct involvement in tissue reconstruction [8]. Clinical trials often use autologous stem cells to treat human diseases without consideration of immune rejection [4]. Unfortunately, patients who may benefit from stem cell therapies often exhibited accompanying systemic diseases, which may compromise the functionalities of their own stem cells and cause uncertainty of autologous transplantation [7]. ASCs have been recognized as a promising stem cell source in recent years because of a series of specific advantages: convenient acquisition of adipose tissue, lack of ethical concerns, and effortless in vitro isolation and expansion [8]. However, poor donor physical conditions (e.g., older age and diabetes) can impair the overall benefits of ASCs and limit their applications [22, 23]. In the present study, we hypothesized that diabetic conditions and aging might change the epigenome, transcriptome, proteome, and metabolome of ASCs. We mainly focused on the transcriptome and conducted RNA-seq to uncover the corresponding underlying mechanisms.

First, we assessed the functional and phenotypic differences of ASCs isolated from diabetic, old, and young donors. The results were generally similar to the findings of previous studies. We found that ASCs isolated from old donors exhibited impaired proliferation and migration abilities; moreover, they easily became senescent after in vitro expansion, as described previously [21]. Previous studies showed that O-ASCs had weakened capacities for accelerating skin wound healing compared to Y-ASCs; similarly, we found that O-ASCs had inferior capacities for modulating HUVEC and fibroblast functions [16]. However, we found that diabetes did not influence the proliferation of ASCs, which considerably differed from the findings in most previous studies [20, 22]. This discrepancy might be related to differences in original sources, and expansion methods, as well as other factors. Importantly, the migratory and senescent phenotypes of diabetic ASCs in our study were consistent with the findings in previous research [22]. Diabetic conditions reportedly impair the therapeutic effects of ASCs in wound healing and critical limb ischemia [19, 44]. Our research also revealed that diabetic ASCs were slightly impaired in their capacities to promote HUVEC angiogenesis and fibroblast migration, which might explain the results of the prior studies. Thus, donor physical conditions must be considered before stem cell therapies.

To the best of our knowledge, the pathogenesis and progression of many diseases (e.g., cancers and metabolic diseases) are often accompanied by the transcriptome alterations. Non-coding RNAs, which are vital components of the transcriptome, have been shown to participate in the pathophysiologic mechanisms underlying those diseases [24, 45]. Previous microarray analyses have identified differences in the expression levels of miRNAs and lncRNAs in BMMSCs isolated from young and old donors [46, 47]. However, the overall ASC transcriptome alterations related to diabetes and aging have not been fully characterized. In the present study, we used RNA-seq to simultaneously explore expression differences in miRNAs, mRNAs, lncRNAs, and circRNAs among ASCs isolated from diabetic, old, and young donors. Our study found that large amounts of RNAs were differentially expressed in Y-ASCs vs. O-ASCs and in D-ASCs vs. O-ASCs. GO analyses showed biological processes such as aging, angiogenesis, cell adhesion, positive regulation of cell proliferation, and cell-cell signaling were both enriched in the two comparisons. Furthermore, the telomere organization term was significantly enriched in GO analyses that compared D-ASCs with O-ASCs. Telomere shortening is recognized as a hallmark of stem cell senescence [48], and a previous study showed that diabetes could significantly affect telomere length [49]. KEGG analyses revealed that the AGE-RAGE signaling, cellular senescence, and p53 signaling pathways were both enriched in the two comparisons. Notably, the p53 signaling pathway reportedly involved in the proliferation, senescence, apoptosis, and differentiation of stem cells [50].

Moreover, we conducted PPI network analyses to further characterize the differentially expressed mRNAs. Comparing Y-ASCs with O-ASCs, we found that MMP3, EGR1, JUNB, BMP4, ITGB8, WNT11, and BMPER were involved in the network; these findings were verified by PCR analyses. A recent study revealed that the induction of EGR1 could trigger glioblastoma cell dedifferentiation into a stem-like state, which involved the expression of pluripotent markers NANOG and OCT4 [51]. JUNB, a member of the AP-1 transcription factor family, has been shown to regulate epidermal stem cells by balancing progenitor proliferation and differentiation [52]. BMP4, a member of the BMP–SMAD signaling axis, could suppress p16/INK4A-mediated cell senescence [53]. In our study, EGR1, JUNB, and BMP4 were all downregulated in O-ASCs, compared with Y-ASCs; this might explain the dysfunction observed in O-ASCs. Additionally, PCR analyses showed that the network members CXCL8, CXCL1, CXCL6, CXCL5, COL18A1, and FMOD were all downregulated in D-ASCs, compared with O-ASCs. These results contrasted markedly from the findings of a previous study, in which ASCs from patients with diabetes or atherosclerosis strongly expressed pro-inflammatory markers such as IL-1β, IL6, and IL-8/CXCL8 [17, 18]. However, a recent study demonstrated that the overexpression of dual chemokines CXCL6 and SDF-1α in ASCs could promote angio-vasculogenesis in those cells [54]. Because these chemokines exhibit multiple functions [55], their specific roles in ASCs require further investigation.

ceRNA networks have been described as an intricate interplay among diverse RNA species, in which mRNAs, lncRNAs, pseudogenes, and circRNAs compete for binding to shared miRNAs [35]. This crosstalk has been shown to participate in many biological processes and human diseases, especially involving oncogenesis and disease progression [56]. However, it has received minimal attention in recent research. A previous study showed that LINC00707 could promote osteogenesis in human BMMSCs by acting as a ceRNA to upregulate WNT2B via miR-370-3p inhibition [57]. circFOXP1 could sustain mesenchymal stem cell identity and differentiation by competing with WNT5A for miR-17-3p/miR-127-5p binding [36]. Because miRNAs are the cores of the ceRNA network, we first evaluated the miRNAs that were differentially expressed in RNA-seq analysis by a secondary PCR analysis approach. Our results revealed that eight miRNAs were differentially expressed between Y-ASCs and O-ASCs. Of these eight miRNAs, miR-210-3p [58] and miR-126 [59] could inhibit apoptosis in ASCs and BMMSCs, while miR-214-3p could anticipate in the therapeutic application of mesenchymal stem cell-derived extracellular vesicles for attenuating radiation-induced lung injury [60]. However, all three miRNAs were downregulated in O-ASCs, which might have contributed to their poor function. Additionally, we found that eight miRNAs were differentially expressed between D-ASCs and O-ASCs. Among these eight miRNAs, miR-302 clusters were strongly expressed in human embryonic stem cells and associated with stem cell pluripotency [61]. Surprisingly, most of the above miRNAs have not been extensively investigated in terms of mesenchymal stem cell phenotypes and functions. Thus, further studies are needed to investigate their characteristics.

Next, we constructed ceRNA networks using bioinformatics methods based on the differentially expressed mRNAs, lncRNAs, circRNAs, and verified miRNAs. The four network maps illustrated extensive potential ceRNA relationships that might play vital roles in ASC biology. Subsets of RNAs in the networks were analyzed by PCR to establish sub-networks. Most involved genes comprised extracellular proteins, and some genes (e.g., COL18A1, MFAP5, ITGB8, FMOD, and PODN) were extracellular matrix-related proteins. SOX4 and ANGPT1, which participate in stemness and angiogenesis, were downregulated in D-ASCs in our study. Liu et al. [62] reported that BMMSCs transfected with ANGPT1 had therapeutic effects on hyperoxia induced optic nerve injury. With respect to non-coding RNAs in the networks, NEAT1 has been proven to act as an oncogene in multiple cancers by binding to various miRNAs [63, 64]. LINC02595 could promote colorectal cancer progression by inhibiting miR-203b-3p activity [65]. Notably, the biological functions of other lncRNAs and circRNAs have not been reported thus far.

The RAET1E-AS1–miR-145-5p–WNT11/BMPER axis was conformed on the basis of ceRNA relationships and expression correlation analyses. miR-145-5p has been recognized as a tumor repressor in many types of cancers [66, 67], a suppressor in the osteogenic differentiation of ASCs [68], and a biomarker for the diagnosis of type 2 diabetes because of its low level in plasma from patients with diabetes [69]. Our study found that the overexpression of miR-145-5p in O-ASCs could rejuvenate cellular senescence and enhance their function. Thus, the modulation of miR-145-5p in ASCs might be a promising method for promoting stem cell function; further investigations are needed to explore the therapeutic effects of modified ASCs in animal models. Chen et al. [70] found that WNT11 overexpression could inhibit ASC proliferation and induce their differentiation into nucleus pulposus cells and osteoblasts. Pérez et al. [71] found that BMPER was upregulated in diet- and obese-derived ASCs; moreover, it could decrease the migration abilities of those cells. However, the role of the lncRNA RAET1E-AS1 in cell biology has not been explored. Further fundamental studies are needed to investigate the specific function of the RAET1E-AS1–WNT11–BMPER network in ASCs and its direct interactions with miR-145-5p.

## Conclusion

**Fig. 9:**
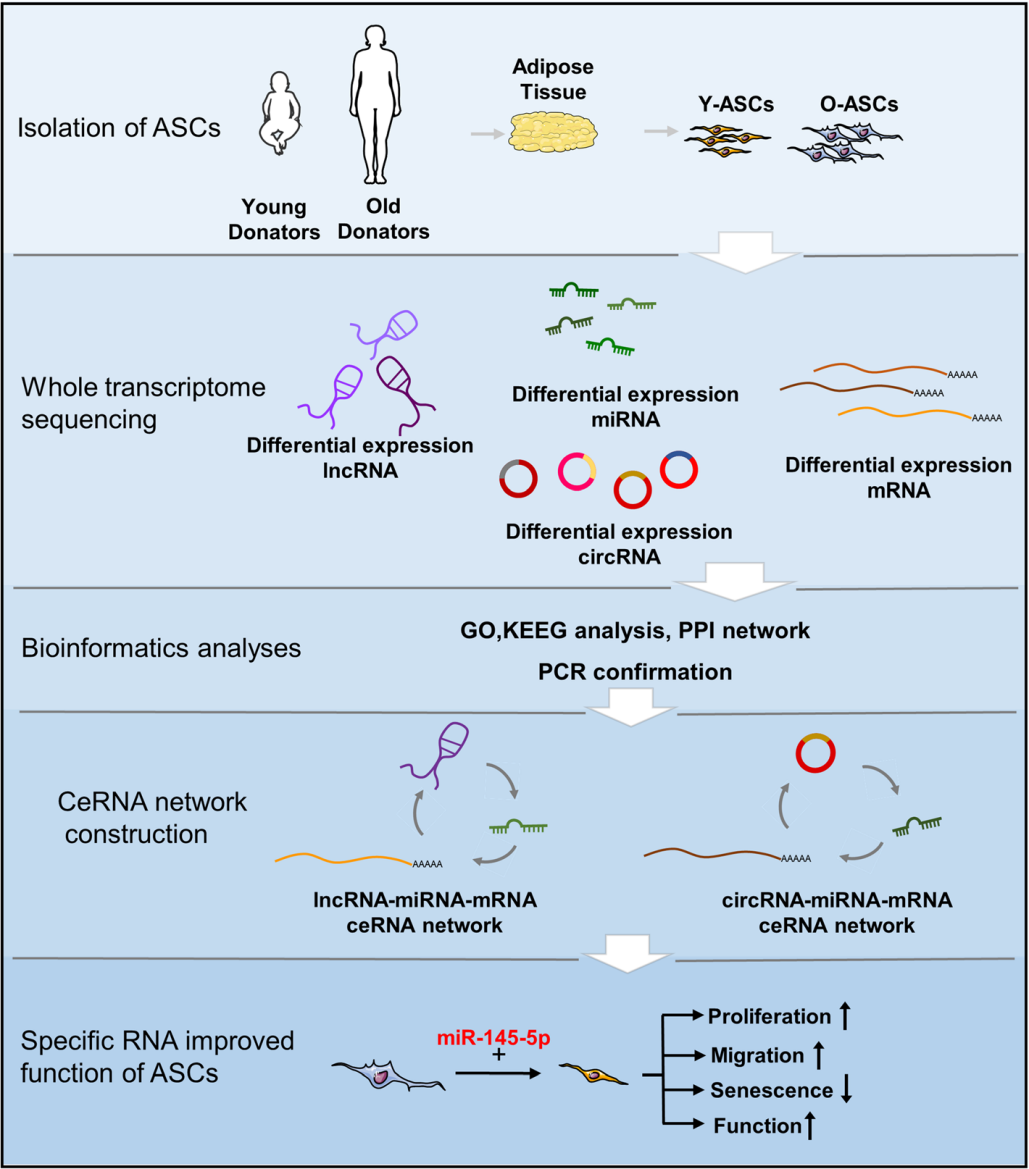
The flow diagram of this study

In the present study, we simultaneously examined changes in the expression patterns of miRNAs, mRNAs, lncRNAs, and circRNAs in ASCs from older donors, younger donors, and donors with diabetes. GO and KEGG pathway analyses were conducted to identify the possible functions of differentially expressed mRNAs. PPI networks were established to find protein genes with critical roles in our disease models. ceRNA networks that included lncRNA–miRNA–mRNA and cirRNA–miRNA–mRNA interactions were successfully constructed based on the bioinformatics analyses and PCR results. Overexpression of miR-145-5p could rejuvenate the O-ASC phenotype and augment their abilities to modulate endothelial cell and fibroblast functions (Fig. 9). Thus, this study may contribute to the broader understanding of the underlying mechanisms of ASC instability and provide novel targets to reverse dysfunction in ASCs isolated from diabetic and old patients.

## Supplementary Information

**Additional file 1: Table S1**–**S4.** Primers used for real-time polymerase chain reaction. **Table S5.** Sequences of mimics and inhibitors.

**Additional file 2: Figure S1.** This figure illustrated the negative results of Fig. 7. The selected mRNAs, lncRNAs and circRNAs without significant difference by PCR analyses were shown in (A, B). (C, D) The correlation analysis found the expression level of miR-145-5p was not significantly correlated with the expression levels of LOC102723591 and hsa_circ_0092630.

**Additional file 3: Data S1.** Differentially expressed miRNAs between O-ASCs and Y-ASCs.

**Additional file 4: Data S2.** Differentially expressed mRNAs between O-ASCs and Y-ASCs.

**Additional file 5: Data S3.** Differentially expressed lncRNAs between O-ASCs and Y-ASCs.

**Additional file 6: Data S4.** Differentially expressed circRNAs between O-ASCs and Y-ASCs.

**Additional file 7: Data S5.** Differentially expressed miRNAs between D-ASCs and O-ASCs.

**Additional file 8: Data S6.** Differentially expressed mRNAs between D-ASCs and O-ASCs.

**Additional file 9: Data S7.** Differentially expressed lncRNAs between D-ASCs and O-ASCs.

**Additional file 10: Data S8.** Differentially expressed circRNAs between D-ASCs and O-ASCs.

**Additional file 11: Data S9.** Differentially expressed miRNAs between D-ASCs, O-ASCs and Y-ASCs.

**Additional file 12: Data S10.** Differentially expressed mRNAs between D-ASCs, O-ASCs and Y-ASCs.

**Additional file 13: Data S11.** Differentially expressed circRNAs between D-ASCs, O-ASCs and Y-ASCs.

**Additional file 14: Data S12.** Differentially expressed lncRNAs between D-ASCs, O-ASCs and Y-ASCs.

**Additional file 15: Data S13.** GO Biological Process Analysis between O-ASCs and Y-ASCs.

**Additional file 16: Data S14.** KEGG Pathway Analysis between O-ASCs and Y-ASCs.

**Additional file 17: Data S15.** GO Biological Process Analysis between D-ASCs and O-ASCs.

**Additional file 18: Data S16.** KEGG Pathway Analysis between D-ASCs and O-ASCs.

**Additional file 19: Figure S2.** The efficiency of mimic and inhibitor transfection were examined. (A, B) Representative images of ASCs treated with 50 nM mimic-NC labeled with cy3 (red) and 200 nM inhibitor-NC labeled with 5-FAM (green). Scale bar = 50 μm. (C, D) The miR-145-5p expression level of ASCs transfected with miR-145-5p mimic or inhibitor was detected by PCR. *n* = 3. ***p < 0.001, ****p< 0.0001.

## Data Availability

Not applicable.

## Abbreviations

AGE: Advanced glycation end products
ANGPT1: Angiopoietin 1
ANK2: Ankyrin 2
APOE: Apolipoprotein E
APOL1: Apolipoprotein L1
ASCs: Adipose-derived stem cells
BMMSCs: Bone marrow mesenchymal stem cells
BMP4: Bone morphogenetic protein 4
BMPER: BMP binding endothelial regulator
C3: Complement C3
CCNA1: Cyclin A1
CCNB1: Cyclin B1
CCND1: Cyclin D1
CDKN2A: Cyclin-dependent kinase inhibitor 2A
ceRNA: Competing endogenous RNA
circRNA: Circular RNA
CLEC3B: C-type lectin domain family 3 member B
COL18A1: Collagen type XVIII alpha 1 chain
CXCL1: C-X-C motif chemokine ligand 1
CXCL12: C-X-C motif ligand 12
CXCL2: C-X-C motif chemokine ligand 2
CXCL5: C-X-C motif chemokine ligand 5
CXCL6: C-X-C motif chemokine ligand 6
CXCL8: C-X-C motif ligand 8
CXCR4: C-X-C motif chemokine receptor 4
EGR1: Early growth response protein 1
FGF: Fibroblast growth factor
FMOD: Fibromodulin
FPKM: Fragments per kilobase of transcript per million fragments sequenced
GBP2: Guanylate binding protein 2
GO: Gene ontology
GPC3: Gypican3
HGF: Hepatocyte growth factor
HIF-1: Hypoxia inducible factor 1 subunit alpha
HLA-F: Major histocompatibility complex, class I, F
HUVECs: Human umbilical vein endothelial cells
IBMIR: Instant blood-mediated inflammatory reaction
IGF1: Insulin-like growth factor 1
IGFBP6: Insulin-like growth factor binding protein 6
IGFBP7: Insulin-like growth factor binding protein 7
IL6: Interleukin 6
ITGB8: Integrin subunit beta 8
KEGG: Kyoto Encyclopedia of Genes and Genomes
lncRNA: Long non-coding RNA
LUC7L3: LUC7 like 3 pre-mRNA splicing factor
MCHR2: Melanin concentrating hormone receptor 2
MFAP5: Microfibril-associated protein 5
miRNA: MicroRNA
MMP9: Matrix metallopeptidase 9
NDUFV3: NADH: ubiquinone oxidoreductase subunit V3
NEAT1: Nuclear paraspeckle assembly transcript 1
NOVA1: NOVA alternative splicing regulator 1
OAS1: 2′-5′-oligoadenylate synthetase 1
OCT4: Organic cation/carnitine transporter4
PDGFA: Platelet-derived growth factor subunit A
PODN: Podocan
PPAR: Peroxisome proliferators-activated receptor
PPI: Protein-protein interaction
QSOX1: Quiescin sulfhydryl oxidase 1
RAET1E-AS1: RAET1E antisense RNA 1
RAGE: Receptor for advanced glycation end products
RARRES3: Retinoic acid receptor responder 3
ROS: Reactive oxygen species
RT-PCR: Real-time polymerase chain reaction
S1PR1: Sphingosine-1-phosphate receptor 1
SLC5A3: Solute carrier family 5 member 3
SOX4: SRY-box transcription factor 4
SPP1: Secreted phosphoprotein 1
TAS2R31: Taste 2 receptor member 31
TNC: Tenascin C
