# Supplementary material for: The whole profiling and competing endogenous RNA network analyses of noncoding RNAs in adipose-derived stem cells from diabetic, old, and young patients
Source: Stem Cell Res Ther. 2021 May 29;12:313. doi: 10.1186/s13287-021-02388-5 (PMC8164820; doi:10.1186/s13287-021-02388-5)
Supplement: Supplementary file 1 — Additional file 1: Table S1–S4. Primers used for real-time polymerase chain reaction. Table S5. Sequences of mimics and inhibitors. [file 13287_2021_2388_MOESM1_ESM.docx]

**Table S1.** miRNAs’ primers used for real-time polymerase chain reaction

| Gene | Primer sequences |
| --- | --- |
| hsa-miR-145-3p  hsa-miR-145-5p  hsa-miR-126-3p  hsa-miR-126-5p  hsa-miR-214-3p  hsa-miR-181a-3p  hsa-miR-210-3p  hsa-miR-766-3p  hsa-miR-204-5p  hsa-miR-370-3p  hsa-miR-543  hsa-miR-153-3p  hsa-miR-299-3p  hsa-miR-485-3p  hsa-miR-193a-3p  hsa-miR-615-3p  hsa-miR-3529-3p  hsa-miR-12136  hsa-miR-302a-3p  hsa-miR-302b-3p  hsa-miR-3074-5p  hsa-miR-29b-1-5p  hsa-let-7i-3p  hsa-miR-337-3p  hsa-miR-328-3p  U6 | Forward 5’-GGATTCCTGGAAATACTGTTCT-3’  Forward 5’-CAGTTTTCCCAGGAATCCCT-3’  Forward 5’-TCGTACCGTGAGTAATAATGCG-3’  Forward 5’-GGGCATTATTACTTTTGGTACGCG-3’  Forward 5’-AGCAGGCACAGACAGGCAGT-3’  Forward 5’-ACCATCGACCGTTGATTGTACC-3’  Forward 5’-CTGTGCGTGTGACAGCGG-3’  Forward 5’-AGCCCCACAGCCTCAGCAA-3’  Forward 5’-TTCCCTTTGTCATCCTATGCCT-3’  Forward 5’-GCTGGGGTGGAACCTGGTAA-3’  Forward 5’-AAACATTCGCGGTGCACTTCTT-3’  Forward 5’-GGGTTGCATAGTCACAAAAGTGATC-3’  Forward 5’-TATGTGGGATGGTAAACCGCTT-3’  Forward 5’-CATACACGGCTCTCCTCTCT-3’  Forward 5’-AACTGGCCTACAAAGTCCCA-3’  Forward 5’-AGCCTGGGTCTCCCTCTTA-3’  Forward 5’-CAACAAAATCACTAGTCTTCCA-3’  Forward 5’-AAGAAAAAGTCATGGAGGCC-3’  Forward 5’-AGTGCTTCCATGTTTTGGTGA-3’  Forward 5’-TAAGTGCTTCCATGTTTTAGTAG-3’  Forward 5’-GTTCCTGCTGAACTGAGCCA-3’  Forward 5’-GCTGGTTTCATATGGTGGTTTA-3’  Forward 5’-GCAAGCTACTGCCTTGCTAA-3’  Forward 5’-CTCCTATATGATGCCTTTCTTC-3’  Forward 5’-CCTCTCTGCCCTTCCGTAAA-3’  Forward 5’-GGAACGATACAGAGAAGATTAGC-3’  Reverse 5’-TGGAACGCTTCACGAATTTGCG-3’ |

**Table S2.** mRNAs’ primers used for real-time polymerase chain reaction

| Gene | Primer sequences |
| --- | --- |
| ITGB8  WNT11  MMP3  EGR1  JUNB  BMPER  BMP4  ANK2  PODN  NOVA1  CLEC3B  RARRES3  IGFBP6  MFAP5  GPRASP1  TEK  JUN  FOS  LRIG3  TGFBR3  ANGPT1  SOX4  SLC5A3  NDUFV3  CXCL8  CXCL1  CXCL6  CXCL5  LUC7L3  FMOD  COL18A1  AHNAK  ABL2  SLC4A7  DST  RIN2  C3  GAPDH  ꞵ-ACTIN | Forward 5’-GGAGGTTTTGACGCCATGCTT-3’  Reverse 5’-CGTCATTGGGCACCACTATGC-3’  Forward 5’-GGGCCAAGTTTTCCGATGCTC-3’  Reverse 5’-CCTGTCTCCCCACTTCACTGTT-3’  Forward 5’-TCCTACTGTTGCTGTGCGTGG-3’  Reverse 5’-CCCGTCACCTCCAATCCAAGG-3’  Forward 5’-CATTTACTCAGCGGCACCCAC-3’  Reverse 5’-AGGTAGTCGGGGATCATGGGA-3’  Forward 5’-GATTGTCCCCAACAGCAACGG-3’  Reverse 5’-TCTTGTGCAGATCGTCCAGGG-3’  Forward 5’-GGGTGCGCTGTGTTGTTCATT-3’  Reverse 5’-CTAAGGTGCTGGGGACAGGAG-3’  Forward 5’-ACATGCGGGATCTTTACCGGC-3’  Reverse 5’-CAGATGTTCTTCGTGGTGGAAGC-3’  Forward 5’-CTCAGTTCCTTGGGCCTGTGA-3’  Reverse 5’-GGTGATGATGCGGCAGATTCG-3’  Forward 5’-GTCCTCATCCTGTCCAGCAACT-3’  Reverse 5’-TCTTCTCCAGCTTGTTGTTCTTG-3’  Forward 5’-CGCATCAAACAAACATTGCCATC-3’  Reverse 5’-GCCCCTGACTGCTCCATTACA-3’  Forward 5’-CCGCATCGCCTACAAGAACTG-3’  Reverse 5’-TTGTCGAACCACTTGCCGTTG-3’  Forward 5’-AGTGCAGAGGTGAAACGGGAG-3’  Reverse 5’-GTCCAAGCTGTTGTTGACCCG-3’  Forward 5’-AGGATGTGAACCGCAGAGACC-3’  Reverse 5’-CTTGGACACCCGCAGAATTGG-3’  Forward 5’-TGCAGTTGTCTAGTCGGGAAGT-3’  Reverse 5’-TTGCTTTGTGGTGGTGGGTTC-3’  Forward 5’-GCCTGAAAAGAAGCCTGGGGA-3’  Reverse 5’-CACCCACTGCACTTGTCTCAAC-3’  Forward 5’-TGTTAGCTTAGGAGGCACACCC-3’  Reverse 5’-CAGTTCAGGGGCTTCTCCAGT-3’  Forward 5’-AGAGGAAGCGCATGAGGAACC-3’  Reverse 5’-TGCGTTAGCATGAGTTGGCAC-3’  Forward 5’-GACTACGAGGCGTCATCCTCC-3’  Reverse 5’-CCGTGGGAATGAAGTTGGCAC-3’  Forward 5’-AGCGCCCATGCCCCACTAC-3’  Reverse 5’-CGAAGGCTTTGAAGGTGGCTCA-3’  Forward 5’-GCAAGGGAGCGTGGATATTGC-3’  Reverse 5’-TACCGCAGCCATTCAGAGGAG-3’  Forward 5’-TCAGTGGCTGCAAAAACTTGAGA-3’  Reverse 5’-CAGCTTTCTGGTCTGCTCTGC-3’  Forward 5’-ACCAACAATGCCGAGAACACG-3’  Reverse 5’-CTCGATCTGCGACCACACCAT-3’  Forward 5’-TGTGGGCCTTCGGGGTTTAAT-3’  Reverse 5’-ATCTGGCCTCCTTGCATCTCC-3’  Forward 5’-TGTCTGCGGAATCAGGGAAGA-3’  Reverse 5’-CTGTGAATAGCACGCTGCCAC-3’  Forward 5’-GCAGTTTTGCCAAGGAGTGCT-3’  Reverse 5’-TTTCTGTGTTGGCGCAGTGTG-3’  Forward 5’-TGCAGGGAATTCACCCCAAGAA-3’  Reverse 5’-GGATGCAGGATTGAGGCAAGC-3’  Forward 5’-GTAAACTGCAGGTGTTCCCCG-3’  Reverse 5’-TTAGAAAAGGGGCTTCCGGGT-3’  Forward 5’-GACCACGCAAGGAGTTCATCC-3’  Reverse 5’-TTCCCGTTCTTCAGGGAGGC-3’  Forward 5’-TGTAGGAGGGATTTCGGCCTG-3’  Reverse 5’-TACAAACGCTCTCGTGGTCCC-3’  Forward 5’-AGCCAGCAGTCCACCTACTAC-3’  Reverse 5’-TACTTCATGCGGGAGGGAACG-3’  Forward 5’-GCCTTGGATTTGGCATGAGGG-3’  Reverse 5’-GTCAAACGGAAACTGCCCTGG-3’  Forward 5’-CACCGTCCTTCGGCATTTCTG-3’  Reverse 5’-TCCCCCACCCTCCATTTTCAC-3’  Forward 5’-ACTTCACTTTGCTGCCTGTGC-3’  Reverse 5’-AGCCTCATTTAGTGCCTGGGG-3’  Forward 5’-TCCATTTGAAGGGGGAAGTCTCT-3’  Reverse 5’-TCTGGTTACTTTGGCATTCTCAGT-3’  Forward 5’-TGAGTGGGGCTCAGATTTGCC-3’  Reverse 5’-TGGAGTGTATCAAGGTGCCGT-3’  Forward 5’-TACTTCCTCCTCCGACTCGCT-3’  Reverse 5’-CTTGGTCACTTTCCCCCTCGT-3’  Forward 5’-AGATGACAGAGGATGCCGTCG-3’  Reverse 5’-TGCTGGGTGTACCCCTTCTTG-3’  Forward 5’-AAAATCAAGTGGGGCGATGCT-3’  Reverse 5’-TGGTTCACACCCATGACGAAC-3’  Forward 5’-CAGCCTTCCTTCCTGGGCAT-3’  Reverse 5’-GGGCAGTGATCTCCTTCTGCAT-3’ |

**Table S3.** lncRNAs’ primers used for real-time polymerase chain reaction

| Gene | Primer sequences |
| --- | --- |
| LOC102723591  RAET1E-AS1  LOC112267883  LINC02595  LOC105373230  NEAT1  LOC101928000  LOC105377989  LOC102724087  LOC100507053  ZEB1-AS1  LOC102724852  LINC01260  LOC107984494  LOC101928570  LOC101929709  LOC105373785 | Forward 5’-TGAACCTGGGCATTCTGGCAC-3’  Reverse 5’-GCCTGGTCTGCCTCCACATC-3’  Forward 5’-TGTCCTTCGGCTTTCTGCTCA-3’  Reverse 5’-TAGAGGCAAGGCCAGGATGTG-3’  Forward 5’-TAGCTGGTTCCAAGTGACCCTC-3’  Reverse 5’-TGACCAGCAAATATCTCCACCCTC-3’  Forward 5’-GGAAATGAGGACCACCGCTC-3’  Reverse 5’-TGGCTTCTACACTGCCACCTG-3’  Forward 5’-CGCATCGGAGTGATCGCTGA-3’  Reverse 5’-CAAATTCTTCCTGTGGCCCCG-3’  Forward 5’-GGGGAAGTAGTCTCGGGTATGC-3’  Reverse 5’-AGGCAATGTGATAGGGGTCGAG-3’  Forward 5’-AGGTGGAGAGTGAGCCCTTAT-3’  Reverse 5’-CAGACCTTGTGAGGCATCTGG-3’  Forward 5’-CCAGGACCCTTGAGACACCTT-3’  Reverse 5’-CACATGGCACTTCTGGAGCAC-3’  Forward 5’-AATTTACTCACGTAGGCCAGGAAT-3’  Reverse 5’-GTTGCCTTGCTCTGGAAGTGTT-3’  Forward 5’-CATTGTGGCCTGTGCTCGAC-3’  Reverse 5’-CTCTTGCAGGGATGCTGGTCT-3’  Forward 5’-ACGGTGTCCTTGCTTTGCTTG-3’  Reverse 5’-GTGGGGTGGGGTCAATTCCAT-3’  Forward 5’-TGGACGTGACAAGCAGGACAT-3’  Reverse 5’-GAAACAGACCCGCTTCTTGCC-3’  Forward 5’-GTAGCTGCCCATATGCCCTCA-3’  Reverse 5’-CCGGCTGAGTTTCTTGCATGG-3’  Forward 5’-ATGTCAGATCAAGGGCCAGGG-3’  Reverse 5’-GCAATCCAGGCCATACGCAAA-3’  Forward 5’-GTGGAGGCTATGAAGATGGAGCA-3’  Reverse 5’-TGAGATGGAGGGGACTTGAGAG-3’  Forward 5’-GGTCTGCTTTCCGACTCCCTT-3’  Reverse 5’-AGGCAGATCCCCCATAACAGC-3’  Forward 5’-TACGATGGGGCGACTCTTGTC-3’  Reverse 5’-GTAACGTGGTCACGGAGCAAC-3’ |

**Table S4.** circRNAs’ primers used for real-time polymerase chain reaction

| Gene | Primer sequences |
| --- | --- |
| hsa_circ_0017534  hsa_circ_0075045  hsa_circ_0080909  hsa_circ_0080906  hsa_circ_0092630  hsa_circ_0088195  hsa_circ_0088199  hsa_circ_0058158  hsa_circ_0013884  hsa_circ_0026497  hsa_circ_0036524 | Forward 5’-GAGCAGCGCATCAGACAGAAC-3’  Reverse 5’-CTGCGGTTGAAGTTGGACACC-3’  Forward 5’-AGCATCCCTGTGGAGGACAAC-3’  Reverse 5’-GAACTGCACCCAGATTCCGC-3’  Forward 5’-TGTGAATCTGGAAAAGGACGCTG-3’  Reverse 5’-AGAATGTGATCTTTGCTTCCCAC-3’  Forward 5’-TTAAAGGCGAGGCTGGTGTGC-3’  Reverse 5’-GTTCTGACCGCATTCCTCTTGG-3’  Forward 5’-ACAGCAGGACCACAATCTCAGT-3’  Reverse 5’-AGGAAAAAGCAGTCACAGTTGC-3’  Forward 5’-CCTGGTGACAGCCAACATCAC-3’  Reverse 5’-GAAGCTCTCCACTTGGGCTGT-3’  Forward 5’-AAGCAAAGGAAGGCGATCCCA-3’  Reverse 5’-AGACCCCTTCATCAGCTGTCC-3’  Forward 5’-TGACACCTGGAGGAGACCACA-3’  Reverse 5’-CTCGCAGTTAAAACCTCGGCT-3’  Forward 5’-GAGAGCCCAACCACTCCTCTG-3’  Reverse 5’-ATTACCAGGGGCAGGTCAAGG-3’  Forward 5’-GATACAGACTGGAGGGCTCGT-3’  Reverse 5’-CGTTACTGTGCCAAGTGGTCG-3’  Forward 5’-TACAGACCCAGAGGGAGCACA-3’  Reverse 5’-GGTAGCTAGTCTGGGAAATCTGGG-3’ |

**Table S5.** Sequences of mimics and inhibitors

| Symbols | Sequences |
| --- | --- |
| hsa-miR-145-5p mimic  mimic NC  hsa-miR-145-5p inhibitor  inhibitor NC | 5’-GUCCAGUUUUCCCAGGAAUCCCU-3’  3’-CAGGUCAAAAGGGUCCUUAGGGA-5’  5’- UUUGUACUACACAAAAGUACUG -3’  3’- AAACAUGAUGUGUUUUCAUGAC -5’  5’-AGGGAUUCCUGGGAAAACUGGAC -3’  5’-CAGUACUUUUGUGUAGUACAAA-3’ |
