# Supplementary figures and images for: The whole profiling and competing endogenous RNA network analyses of noncoding RNAs in adipose-derived stem cells from diabetic, old, and young patients
Source: Stem Cell Res Ther. 2021 May 29;12:313. doi: 10.1186/s13287-021-02388-5 (PMC8164820; doi:10.1186/s13287-021-02388-5)

**A**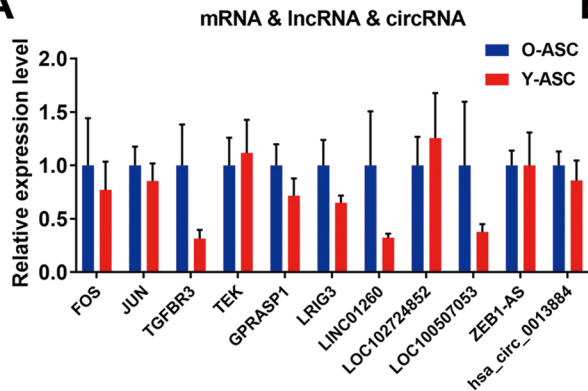**B**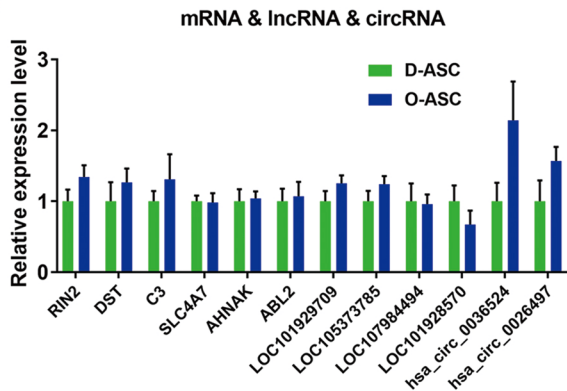**C**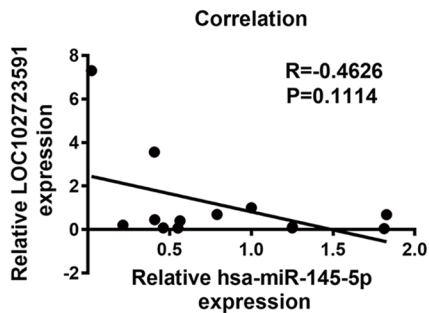**D**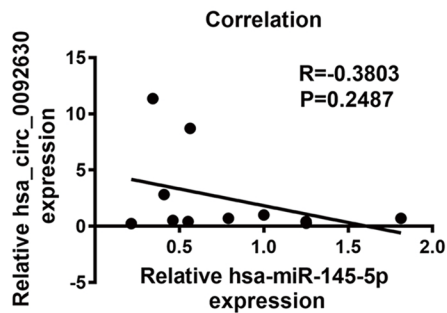

Supplement: Supplementary file 2 — Additional file 2: Figure S1. This figure illustrated the negative results of Fig. 7. The selected mRNAs, lncRNAs and circRNAs without significant difference by PCR analyses were shown in (A, B). (C, D) The correlation analysis found the expression level of miR-145-5p was not significantly correlated with the expression levels of LOC102723591 and hsa_circ_0092630. [file 13287_2021_2388_MOESM2_ESM.pdf]

**A**

mimic-NC-cy3 50nM

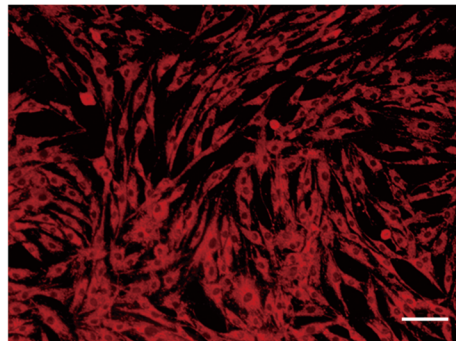**B**

inhibitor-NC-5-FAM 200nM

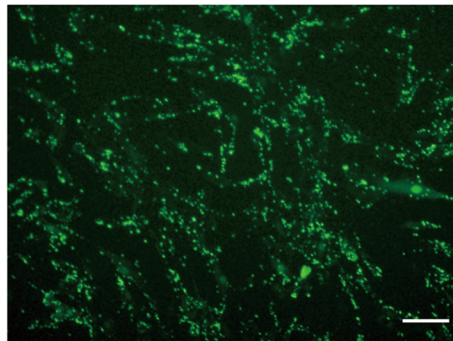**C**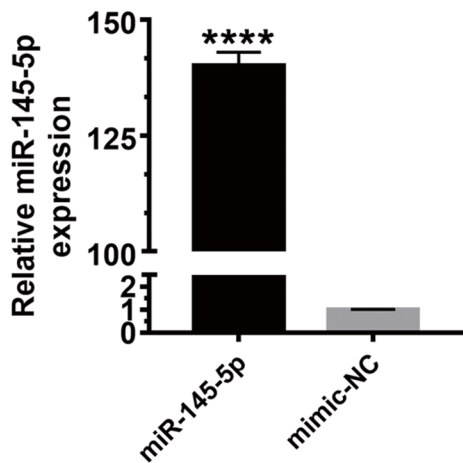**D**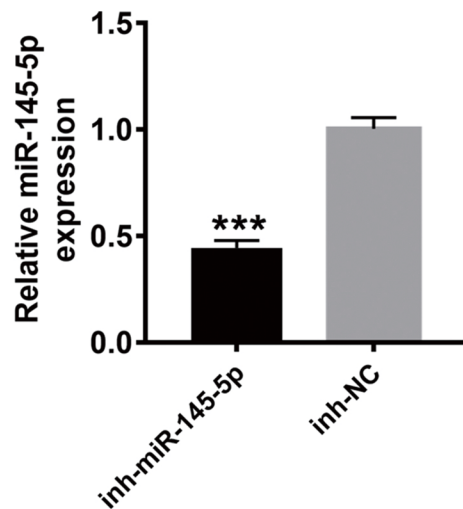

Supplement: Supplementary file 19 — Additional file 19: Figure S2. The efficiency of mimic and inhibitor transfection were examined. (A, B) Representative images of ASCs treated with 50 nM mimic-NC labeled with cy3 (red) and 200 nM inhibitor-NC labeled with 5-FAM (green). Scale bar = 50 μm. (C, D) The miR-145-5p expression level of ASCs transfected with miR-145-5p mimic or inhibitor was detected by PCR. n = 3. ***p < 0.001, ****p< 0.0001. [file 13287_2021_2388_MOESM19_ESM.pdf]
